# A data-driven evaluation of the size and content of expanded carrier screening panels

**DOI:** 10.1038/s41436-019-0466-5

**Published:** 2019-02-28

**Authors:** Rotem Ben-Shachar, Ashley Svenson, James D. Goldberg, Dale Muzzey

**Affiliations:** Myriad Women’s Health, Inc. (formerly Counsyl, Inc.), South San Francisco, CA USA

**Keywords:** genetic testing, expanded carrier screening, clinical utility, clinical guidelines, clinical detection rate

## Abstract

**Purpose:**

The American College of Obstetricians and Gynecologists (ACOG) proposed seven criteria for expanded carrier screening (ECS) panel design. To ensure that screening for a condition is sufficiently sensitive to identify carriers and reduce residual risk of noncarriers, one criterion requires a per-condition carrier rate greater than 1 in 100. However, it is unestablished whether this threshold corresponds with a loss in clinical detection. The impact of the proposed panel design criteria on at-risk couple detection warrants data-driven evaluation.

**Methods:**

Carrier rates and at-risk couple rates were calculated in 56,281 patients who underwent a 176-condition ECS and were evaluated for panels satisfying various criteria. Condition-specific clinical detection rates were estimated via simulation.

**Results:**

Different interpretations of the 1-in-100 criterion have variable impact: a compliant panel would include between 3 and 38 conditions, identify 11–81% fewer at-risk couples, and detect 36–79% fewer carriers than a 176-condition panel. If the carrier rate threshold must be exceeded in all ethnicities, ECS panels would lack prevalent conditions like cystic fibrosis. Simulations suggest that the clinical detection rate remains >84% for conditions with carrier rates as low as 1 in 1000.

**Conclusion:**

The 1-in-100 criterion limits at-risk couple detection and should be reconsidered.

## INTRODUCTION

Carrier screening facilitates reproductive decision-making by identifying couples at risk for conceptuses affected with autosomal recessive (AR) and X-linked conditions.^1^ Advances in genomic technology coupled with decreasing sequencing costs have led to the advent and adoption of expanded carrier screening (ECS) for tens to hundreds of recessive conditions simultaneously.^2^ For AR conditions, a couple is at risk if both individuals are carriers of the same condition, with the conceptus having a 25% probability of being affected with the condition. For X-linked conditions, a couple is at risk if the female is a carrier: the probability of a male conceptus being affected with the condition can be up to 50% (ref. ^3^). The American College of Obstetricians and Gynecologists (ACOG) states that ECS is an acceptable strategy for carrier screening.^1^ However, no consensus exists on which conditions should be included on an ECS panel.

Rather than prescribing specific conditions for an ECS panel, professional societies have provided general guidelines for ECS panel design, stressing that panels should maximize clinical utility and not simply follow the model of “the more conditions, the better.”^1,4,5^ Yet there remains no recommended set of ECS conditions in part because there are over 1000 possible single-gene recessive conditions that could be included on a panel,^6^ and it is difficult to determine unambiguous criteria.^7^ As a result, the size and content of ECS panels vary widely across laboratories.^7,8^ In a recent study comparing 16 commercially available ECS offerings, panel size ranged from 41 to 1792 conditions, with only 3 conditions screened by all panels.^7^

Discrepancies in panel size and content have led to a growing desire for guidelines that delineate which conditions should be included on ECS panels.^1,8^ Because ECS is commonly performed sequentially (i.e., ECS on initial patient and single-gene screening as needed on the partner), inclusion of each additional condition on a panel poses a trade-off: identification of at-risk couples provides actionable information to guide pregnancy management,^9^ while concomitant detection of carriers can increase patient anxiety,^2,10,11^ require additional counseling,^2,10^ and introduce logistical burden for the provider.^2,11,12^ For many conditions, the benefit of identifying at-risk couples offsets these challenges, but it is unclear when a condition is too rare to warrant screening. To that end, ACOG^1^ and a clinical assessment of ECS panel design^8^ recommend a 1-in-100 carrier rate threshold for condition inclusion. However, these studies do not quantitatively consider how the 1-in-100 carrier-rate threshold affects the trade-off between carrier identification and at-risk couple identification.

Professional societies further emphasize that for an ECS panel to have high clinical utility, conditions on ECS panels should minimize residual risk (i.e., the risk that a patient carries a pathogenic variant after screening negative).^1,3,4,8^ Genetics professionals recommend that minimal residual risk be achieved by setting a minimum threshold for screening detection rate,^8^ which depends on two compounding factors: analytical detection rate (the ability to accurately detect variants) and clinical detection rate (the ability to accurately determine if a variant is pathogenic or benign). High analytical detection rates have been demonstrated for most conditions on ECS panels,^8,13^ but clinical detection rates have yet to be systematically evaluated.

Building on the guidance of professional societies, a study proposed and applied panel design criteria to seven commercially available ECS offerings, yielding a panel of 96 conditions.^8^ A complementary approach proposed an algorithm to classify condition severity based on disease characteristics^14^ and developed a methodology to maximize detection of conceptus disease risk while ensuring accurate variant interpretation, culminating in a 176-condition panel.^15^ The nearly twofold disparity in panel size resulting from these two approaches, both of which applied principled panel design criteria, underscores the need for greater clarity and objectivity in guidelines.

Given the importance of ensuring that ECS panels maximize clinical utility, a data-driven approach is needed to evaluate the impact of professional society condition inclusion criteria on detection of carriers and at-risk couples. We evaluated the guidelines for ECS panel design from ACOG’s 2017 committee opinion,^1^ which recommended that each ECS condition meet several of seven proposed criteria: (1) have a well-defined phenotype, (2) have a detrimental effect on quality of life, (3) cause cognitive or physical impairment, (4) require surgical or medical intervention, (5) have an onset early in life, (6) have prenatal diagnosis available, and/or (7) have a 1-in-100 or greater carrier rate. We retrospectively analyzed data from a panethnic cohort of over 50,000 patients screened with a 176-condition ECS panel and evaluated how exclusion of conditions that did not meet criteria impacted detection of carriers and at-risk couples. We show that all definitions and applications of the 1-in-100 carrier rate threshold limits detection of at-risk couples, and different definitions cause at-risk couple detection rates to vary by 11-fold. Instead of a carrier rate threshold, we propose an alternative measure—estimation of a clinical detection rate—to evaluate when a condition is too rare to be included in an ECS panel by quantifying if there is enough evidence to determine the clinical association between detected variants and disease.

## MATERIALS AND METHODS

### Cohort design

We retrospectively analyzed de-identified data from patients who underwent ECS over a 17-month period using a next-generation sequencing-based 176-condition panel that includes (1) detection of novel single-nucleotide variants (SNVs) and short insertions and deletions (indels) in full exons and regions of introns associated with disease, (2) panel-wide identification of copy-number variants, and (3) specialized assays for technically challenging genes (Foresight; Myriad Women’s Health, South San Francisco, CA).^15,16^ We constructed two patient cohorts for different purposes: one based on individual patients and used for estimates representative of the US population, and another that focused on couples for empirical analysis.

The first patient cohort, consisting of 56,281 patients and used for the majority of analyses, excluded patients who had a family or personal history of disease or reported consanguinity. The majority of patients in the cohort were female (70%, *N* = 39,454), with 50% (*N* = 19,826) indicating they were pregnant when undergoing ECS (an underestimate because pregnancy status is requested but not required). The cohort was representative of a wide range of self-reported ethnicities (Table S1). For some analyses, to reflect the general US population, we weighted ethnicity-specific carrier rates and at-risk couple rates by ethnicity-specific frequencies gathered from US census data (Table S1). Unless otherwise specified, ‘‘carrier rate’’ describes the US-weighted carrier rate.

The second cohort exclusively included couples who received ECS to enable calculation of the empirical frequencies of at-risk couples per condition. For most analyses of at-risk couples, couples with disease history or consanguinity were excluded, resulting in 8736 couples among whom 314 were at risk. Figure S1 includes all couples who underwent ECS (*N* = 11,536) and were identified as at risk (N = 501; ethnicity-specific frequencies in Table S1).

This study was reviewed and designated as exempt from institutional review board (IRB) oversight (as granted by Western IRB on 23 April 2018). All patients provided informed consent for testing and anonymized research.

### ECS condition classification using ACOG guidelines

For each of the 176 conditions on the ECS panel, we evaluated which of the seven ACOG criteria (see “Introduction”) were met (Table S2). These criteria were evaluated by a certified genetic counselor, using refined classification criteria described in Supplementary Text S1.

We evaluated the criterion suggesting that a condition have a 1-in-100 or greater carrier rate in the following ways: (1) a condition has a 1-in-100 or greater carrier rate in any ethnicity, (2) a condition has a 1-in-100 or greater carrier rate when ethnicities are weighted by their US census frequencies, or (3) a condition has a 1-in-100 or greater carrier rate in all ethnicities (Table S3). We considered two carrier rate thresholds for X-linked conditions: (1) a carrier rate threshold of 1 in 100 and (2) a carrier rate threshold of 1 in 10,000, which resembles a 1-in-100 carrier rate for AR conditions by yielding a prevalence of 1 in 40,000 (Table S3). Descriptions of how carrier and at-risk couple rates were computed are provided in Supplementary Text S2.

### Modeling a clinical detection rate

Clinical detection rate is a function of variant frequencies and the ability to classify identified variants as being pathogenic or benign. Given our large cohort, we assume that the majority of pathogenic variants for each condition have been observed—consistent with a recent study^17^—and that their frequencies are empirically determined from our data set. However, we also presume that some rare pathogenic variants have not been observed in our data set. We account for these unobserved variants by assuming (1) that the number of unobserved pathogenic variants in our cohort is proportional to the number of observed pathogenic variants and (2) that the frequency of unobserved variants reflects the frequency of the least-common observed pathogenic variant (Supplementary Text S3) (ref. ^17^). To model whether a variant can be classified correctly as pathogenic, we assumed that a minimum of three or more literature-reported cases are needed, consistent with American College of Medical Genetics and Genomics (ACMG) variant classification guidelines that stress the importance of case reports when assessing variant pathogenicity.^18^

For each condition, we determined the expected number of literature-reported cases worldwide based on the world population size, US-weighted carrier rates, and the expected percentage of literature-reported cases (Supplementary Text S3). Given the expected number of reported cases and the relative pathogenic variant frequencies for each condition, we simulated the number of reported cases for each observed variant, assuming that all unobserved variants will have no reported cases. We define the estimated clinical detection rate as the sum of adjusted variant frequencies among variants with three or more simulated reported cases (Supplementary Text S3). We repeated the simulations of variant-specific case reports for each condition 10,000 times. All analyses were performed using Python 2.7.10, Numpy 1.13.1, and Pandas 0.20.3.

## RESULTS

### Quantifying impact of ACOG guidelines

We retrospectively analyzed an ethnically diverse cohort of 56,281 average-risk patients who underwent ECS with a 176-condition panel to determine how proposed ACOG panel design criteria would have impacted the detection rates of at-risk couples and carriers. We further determined how many empirically observed at-risk couples would not have been identified if proposed guidelines were strictly followed.

**Fig. 1:**
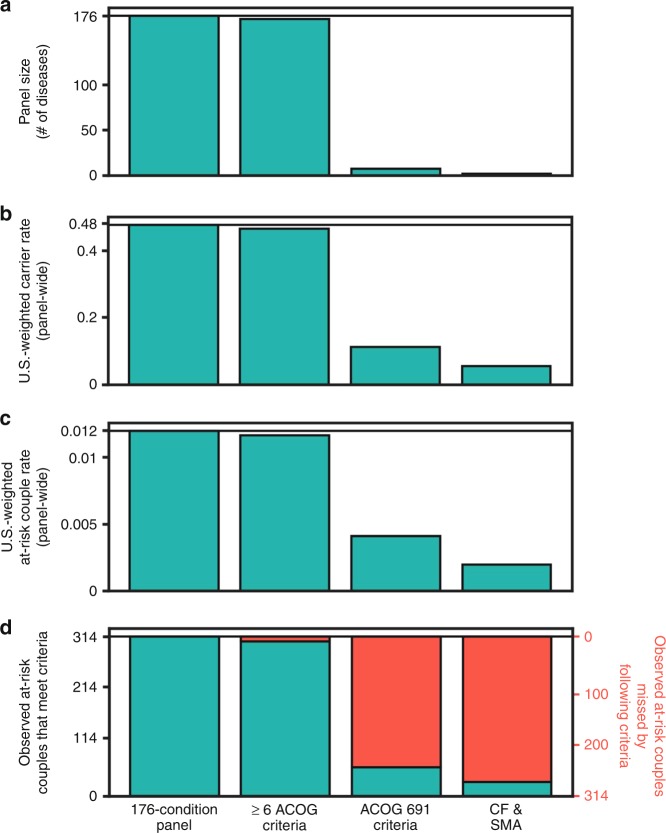
**Impact of American College of Obstetricians and Gynecologists (ACOG) guidelines on panel size, carrier rates, and at-risk couple rates.** We consider four panels: the full 176-condition panel, the subset of conditions that meet the first six ACOG criteria (excluding the 1-in-100 criteria), the subset of conditions that meet the ethnicity-specific requirements in ACOG guidelines 691 (ref. ^19^), and a panel that includes only cystic fibrosis (CF) and spinal muscular atrophy (SMA). (**a**) The number of diseases that meet criteria. (**b**) U.S.-weighted panel carrier rates. (**c**) US-weighted at-risk couple rates. (**d**) The number of observed at-risk couples that would be identified (green) or omitted (red) by the indicated panel. Specific conditions of observed at-risk couples are shown in Figure S1. Horizontal lines show respective numbers for the 176-condition panel.

We evaluated the collective impact of six ACOG criteria unrelated to carrier rate on a panel’s at-risk couple rate and the panel carrier rate, which we define as the proportion of patients in the cohort who were carriers of at least one condition on the panel. Of the 176 conditions on the ECS panel, 172 met all six criteria, with the remaining 4 conditions not present in childhood in the majority of affected patients (Fig. 1a). Limiting an ECS panel to these 172 conditions would reduce the panel carrier rate, the at-risk couple rate, and the number of observed at-risk couples each by 3% (Fig. 1b–d). We compared this subpanel with a panel that meets the ethnicity-specific screening guidelines outlined in ACOG committee opinion 691 (denoted “ACOG 691”)^19^ and a panel that includes only cystic fibrosis (CF) and spinal muscular atrophy (SMA), the two conditions for which ACOG specifically recommends screening in all ethnicities (denoted “CF/SMA”).^19^ For the ACOG 691 panel, carrier identification would be reduced by 77%, at-risk couple identification would be reduced by 66%, and 258 observed at-risk couples (82%) would not have been identified (Fig. 1b–d). For the CF/SMA panel, carrier identification would be reduced by 88%, at-risk couple identification would be reduced by 84%, and 286 observed at-risk couples (91%) would not have been identified (Fig. 1b–d).

### Large variability in ethnicity-specific carrier rates

**Fig. 2:**
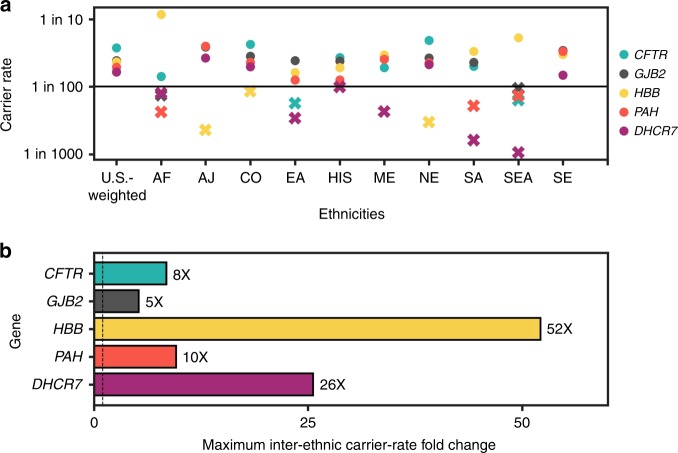
**Carrier rates vary widely by ethnicity.** (**a**) Self-reported ethnicity-specific carrier rates for the five conditions with the highest US-weighted carrier rates in the 176-condition panel for which an ethnicity-specific carrier rate is less than 1 in 100 in at least one ethnicity. Ethnicities with carrier rates below a 1-in-100 carrier rate are denoted with Xs; ethnicities with carrier rates above the 1-in-100 carrier rate are denoted with circles. (**b**) The relative difference in carrier rates between the highest and lowest ethnicity-specific carrier rates for each condition in (**a**). *CFTR* cystic fibrosis, *GJB2* GJB2-related DFNB1 nonsyndromic hearing loss and deafness, *HBB* Hb β chain-related hemoglobinopathy, *PAH* phenylalanine hydroxylase deficiency, *DHCR7* Smith–Lemli–Opitz syndrome. AF African, AJ Ashkenazi Jewish, CO Caucasian/other, EA East Asian, HIS Hispanic, ME Middle Eastern, NE Northern European, SA South Asian, SE Southern European, SEA Southeast Asian.

To determine the impact of the final ACOG criterion, that ECS conditions have carrier rates higher than 1 in 100, we examined the variation in ethnicity-specific carrier rates. We found wide variability in ethnicity-specific carrier rates for five prevalent conditions for which one or more ethnicities have a carrier rate below 1 in 100 (Fig. 2a). The relative difference between the maximum and minimum ethnicity-specific carrier rates ranged from 5-fold to 52-fold across conditions (Fig. 2b). Even for prevalent conditions such as cystic fibrosis, for which panethnic screening is recommended,^19^ multiple ethnicities have a carrier rate below 1 in 100 (Fig. 2a).

### Any interpretation of the 1-in-100 carrier rate criterion limits detection of at-risk couples

**Fig. 3:**
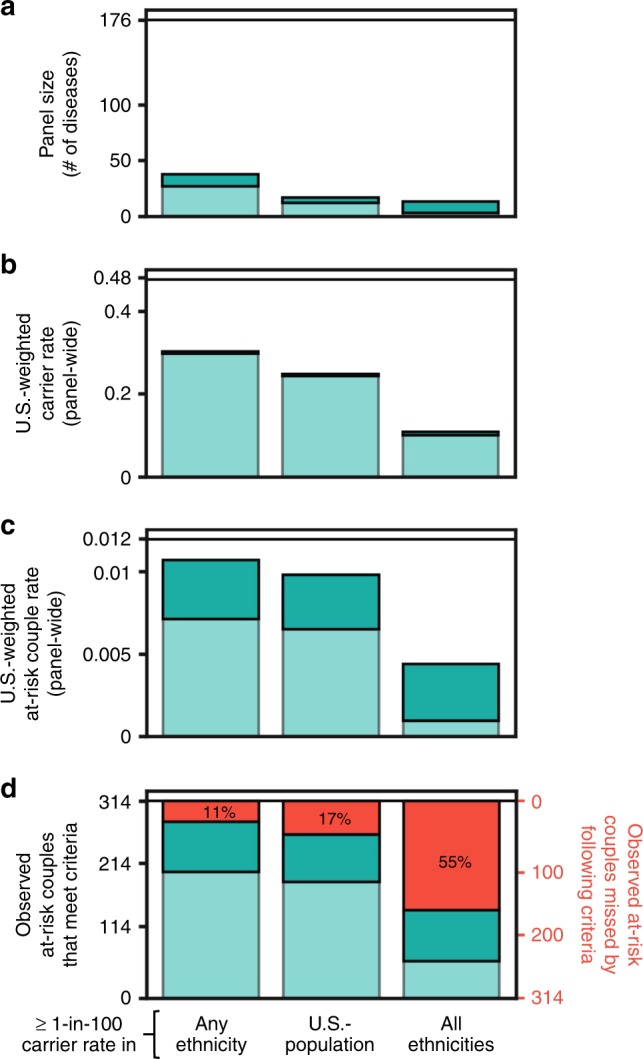
**Impact of different 1-in-100 carrier rate threshold definitions on carrier rates and at-risk couple rates.** Included conditions met the first six American College of Obstetricians and Gynecologists (ACOG) criteria (Fig. 1) and different definitions of the 1-in-100 carrier rate ACOG criteria (*x*-axis). Shading of green bars denotes different carrier rate thresholds for X-linked conditions: a carrier rate threshold of 1 in 100 (light green) and a carrier rate threshold of 1 in 10,000 (dark green). (**a**) The number of diseases that meet criteria. (**b**) US-weighted panel carrier rates. (**c**) US-weighted at-risk couple rates. (**d**) The number of observed at-risk couples that would be identified by the panel subset. Specific conditions of observed at-risk couples are shown in Figure S1. Horizontal lines show respective numbers for the 176-condition panel.

Different interpretations of the 1-in-100 carrier rate threshold impact identification of carriers and at-risk couples. We quantified the impact of three threshold definitions, ranked from least stringent to most stringent: 1-in-100 carrier rate in any ethnicity, 1-in-100 US-weighted carrier rate, and 1-in-100 carrier rates in all ethnicities. We further stratified these three definitions by two carrier rate thresholds for X-linked conditions (see “Materials and methods”). A compliant panel would reduce the panel size from 176 to between 3 and 38 conditions, depending on the definition used (Fig. 3a). Carrier rates would be reduced between 36% and 79%, at-risk couple rates would be reduced between 11% and 92%, and between 33 (11%) and 255 (81%) observed at-risk couples would not be identified (Fig. 3b–d). These data show that any interpretation of the 1-in-100 carrier rate threshold criteria limits detection of at-risk couples relative to the 176- condition panel and that the extent of reduction varies widely.

### Panel carrier rates and at-risk couple rates saturate at large panel sizes

**Fig. 4:**
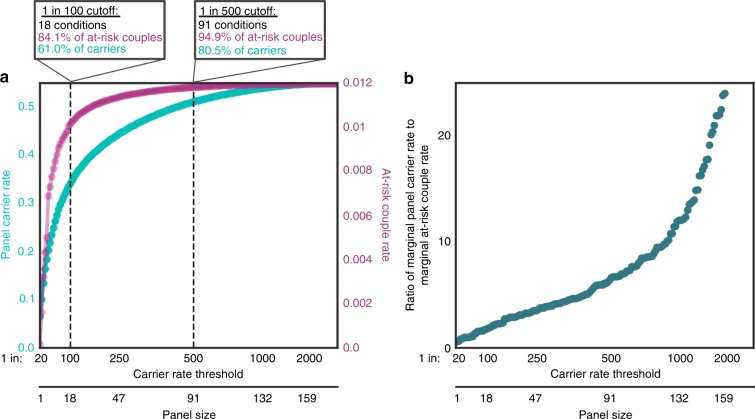
**The relationship between panel size, panel carrier rate, and at-risk couple rate.** (**a**) The panel carrier rate (green) and panel at-risk couple rate (purple) are plotted as a function of the carrier rate threshold and panel size (*x*-axis). Conditions are ordered from most to least prevalent based on US-weighted carrier rate. Because X-linked and autosomal recessive (AR) conditions are inherited differently, carrier rates for X-linked conditions were transformed to corresponding AR carrier rates based on their at-risk couple rates. For example, for an X-linked condition with a carrier rate and at-risk couple rate of 1 in 10,000, the carrier rate would be transformed to 1 in 100. (**b**) Ratio of marginal panel carrier rate to marginal at-risk couple rate as a function of the panel size determined by carrier rate threshold (*x*-axis). The panel carrier rate was calculated from condition-specific US-weighted carrier rates. At-risk couple rates were calculated as the square of the US-weighted carrier rate.

Because detecting at-risk couples incurs costs associated with identification of carriers, we sought to understand the quantitative interplay between the panel carrier rate and the at-risk couple rate as a function of panel size and US-weighted carrier rates. The panel carrier rate increases as more conditions are added to a panel; however, this increase begins to saturate as rare conditions are added because many carriers of a rare condition were already carriers of at least one common condition (Fig. 4a). Growth in the panel carrier rate is rapid for panels with fewer than 18 conditions, where condition-specific carrier rates exceed 1 in 100. Even though a panel with 18 conditions has nearly 10 times fewer genes than the 176-gene panel, this small panel identifies 61.0% of the carriers discovered on the large panel. Adding 73 more conditions to the panel, corresponding to a carrier rate threshold of 1 in 500, identifies 80.5% of panel carriers. The remaining 85 rare conditions that complete the 176-condition panel would increase the panel carrier rate by 19.5%.

Detection of at-risk couples also increases as conditions are added to a panel and saturates as rare conditions are added to the panel (Fig. 4a). However, the at-risk couple rate saturation occurs at smaller panel sizes than the panel carrier rate because at-risk couple detection is proportional to the square of the condition carrier rate. A panel with 18 conditions accounts for 84.1% of at-risk couples. The addition of 73 conditions to the panel increases the percentage of at-risk couples identified to 94.9%, and the remaining 85 rare conditions increase the percentage of at-risk couples identified by 5.1% (Fig. 4a).

We reasoned that a well-motivated carrier rate threshold would occur when the marginal cost of screening a condition disproportionately outweighed its marginal benefit. As such, we viewed detection of at-risk couples as the benefit of carrier screening, considered identification of carriers as the cost, and quantified the ratio of marginal panel carrier rate to the marginal at-risk couple rate (Fig. 4b). This clinically focused cost-to-benefit ratio grows as conditions become rarer, but the relationship is roughly linear down to carrier rates as low as 1 in 1000, where a subtle inflection point appears (Fig. 4b). Notably, however, no conspicuous change in the ratio near a frequency of 1 in 100 is apparent.

### Estimated clinical detection rate may provide an alternative for determining ECS panel rare disease exclusion criteria

**Fig. 5:**
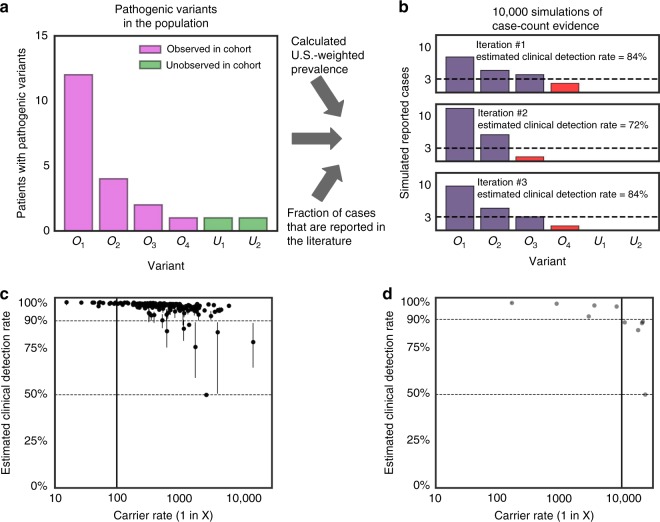
**Estimation of clinical detection rate.** (**a**, **b**) A model schematic showing how clinical detection rate is estimated for a hypothetical autosomal recessive (AR) condition. We assume a condition has six pathogenic variants and a carrier rate of 1 in 10,000, resulting in a prevalence of 1 in 400,000,000. (**a**) Assumed number of pathogenic variants, including both observed variants (purple, variants denoted with O) and a minority of unobserved variants (green, variants denoted with U). (**b**) Simulations of the expected number of reported cases (assuming all cases will be reported). The estimated clinical detection rate is defined as the sum of the variant frequencies for variants that can be classified as pathogenic, determined by three or more estimated case reports (shown in blue). Variants whose pathogenicity cannot be determined are shown in red or have no reported cases. (**c**, **d**) Estimated clinical detection rates for (**c**) AR conditions and (**d**) X-linked conditions on the 176-condition panel. US-weighted carrier rates and estimated clinical detection rate for each condition are shown when three reported cases are needed to determine pathogenicity for each variant. Dots show median estimated clinical detection rate from 10,000 iterations per condition, and lines show corresponding 95% confidence intervals. We excluded X-linked severe combined immunodeficiency and X-linked ornithine transcarbamylase deficiency from this analysis because we did not observe any carriers of these X-linked conditions during the study period (see Supplementary Text S3). Conditions and corresponding clinical detection rate estimates are provided in Table S4.

Identification of at-risk couples and reduction in residual risk for patients who screen negative can only occur if the clinical detection rate of a condition is high. Because direct measurement of clinical detection rate is challenging for rare conditions, we developed a statistical framework that estimates clinical detection rate by modeling whether a sufficient number of cases have been reported in the literature to interpret the pathogenicity of observed variants (see “Materials and methods”). A schematic of the methodology is shown in Fig. 5a, b.

Generally, the estimated clinical detection rate was lower for rare conditions than for common conditions, consistent with rare conditions having fewer reported cases than common conditions (Fig. 5c, d). However, a rare condition with a small number of recurrent pathogenic variants may have higher estimated clinical detection rate than a more common condition with a large number of low-frequency pathogenic variants. For example, delta-sarcoglycanopathy (*SGCD*) has a carrier rate of 1 in 6000 but only four observed pathogenic variants, and its median estimated clinical detection rate (from 10,000 simulated iterations) was 98%. By contrast, methylmalonic acidemia, cblA-type, (*MMAA*) has a carrier rate of 1 in 600 and 13 observed pathogenic variants, giving a median estimated clinical detection rate of 85%.

Each AR condition that had a carrier rate of 1 in 100 or greater (13 conditions) had an estimated clinical detection rate above 97% (Fig. 5c). With the exception of *MMAA* described above, the remaining 123 conditions with carrier rates above 1 in 1000 had a median estimated clinical detection rate above 90% (Fig. 5c), with 109 (88%) conditions having a median estimated clinical sensitivity above 97%. For the remaining 50 conditions with carrier rates below 1 in 1000, 39 conditions had median estimated clinical detection rate above 90% (Fig. 5c, d), suggesting that residual risk reduction is possible for many rare conditions. Furthermore, these estimates are robust to adjustments of the assumed number of reported cases needed to interpret variant pathogenicity (Figures S4–S6) and the assumed number of unobserved variants (Figure S7). In sum, we demonstrate that diseases with carrier rates well below 1 in 100 achieve greater than 90% median estimated clinical detection rate.

## DISCUSSION

Precise and well-motivated panel content criteria are needed to ensure that ECS results maximize detection of at-risk couples and facilitate reproductive decision-making.^1,3,5^ Here, we have shown the first data-driven evaluation, to our knowledge, of the impact of ACOG guidelines^1,5^ for ECS panel content on detection of carriers and, critically, at-risk couples. Our analysis leveraged screening results from a diverse cohort of over 50,000 average-risk patients screened for 176 recessive conditions.

Quantitative inclusion criteria encourage consistency in ECS offerings because numbers are unambiguous; however, the choice of which numbers to use should be transparently data-driven, such that the implications of guidelines are clear. The 1-in-100 carrier rate threshold for condition inclusion proposed by ACOG aimed to address a trade-off between achieving high clinical utility and minimizing anxiety, but, importantly, data were not presented to support this particular threshold.^1^ Stevens et al. supported this threshold because a woman who screens positive for a recessive condition with a carrier rate of less than 1 in 100 would have a reproductive risk of less than 1 in 400, similar to risk cutoffs for common prenatal screening tests such as maternal serum screening.^8^ However, their justification of this threshold is unfounded because it is based on an unintended use of ECS (the testing of a single patient) rather than the intended use (the sequential testing of a couple, supported by medical guidelines^1,19^). When used as intended, a woman who screens positive for a condition with carrier rate of 1 in 100 would likely not need to proceed immediately to diagnostic testing because she can refine her residual risk through testing of her partner: if he screens positive, the reproductive risk is 1 in 4; if he screens negative, the risk can be as low as 1 in 250,000 (ref. ^20^).

We directly evaluated the clinical impact of the 1-in-100 carrier rate threshold for inclusion of conditions on ECS panels and found that it warrants revisiting. The criterion does not specify in which ethnicities the 1-in-100 threshold should be satisfied, and does not offer guidance for X-linked conditions, which contribute disproportionately to at-risk couple detection compared with AR conditions with similar carrier rates. Critically, our analysis demonstrated that any definition of the 1-in-100 carrier rate threshold limits detection of at-risk couples.

We introduced the concept of a “panel carrier rate”—defined as the proportion of patients who were carriers of at least one condition on the panel—because it describes how frequently single-gene partner testing is needed in a sequential-screening workflow and, thus, reflects the logistical and economic costs incurred to reap the clinical benefit of identifying at-risk couples. Our analysis showed that a 1-in-100 carrier rate threshold was not a conspicuously clear choice based on the data: both the panel carrier rate and at-risk couple rate saturate as rare diseases are included in ECS panels (Fig. 4a), yet this saturation occurs beyond the 1-in-100 carrier rate threshold for both panel carrier rates and at-risk couple rates. We additionally saw no clear point at which the marginal cost of screening and detecting carriers far exceeded the marginal benefit of detecting at-risk couples (Fig. 4b). Therefore, the clinical burden associated with identification of carriers and testing of their partners is not substantially reduced by excluding rare conditions from an ECS panel. Taken together, these results show that the 1-in-100 carrier rate threshold will limit detection of at-risk couples without substantially reducing clinical burden.

Despite the drawbacks of the 1-in-100 criterion, it is important to determine when a condition is too rare to include on an ECS panel. For instance, if a condition is so rare that the pathogenicity of variants cannot be interpreted, then the test will have a 0% clinical detection rate, rendering screening useless. All conditions on an ECS panel should have a high analytical detection rate and clinical detection rate that together minimize residual risk.^3,8^ Because the analytical detection rate is >99.9% for most conditions,^13^ we suggest that ECS panel content criteria should focus on defining an acceptable clinical detection rate. A clinical detection rate threshold would directly measure variant interpretability and indirectly correspond to disease prevalence, whereas a carrier rate threshold alone does not capture variant interpretability. We developed a statistical method to estimate clinical detection rates for conditions on the 176-condition panel and demonstrated that conditions with carrier rates as low as 1 in 1000 have a greater than 84% estimated clinical detection rate (Fig. 5). Clinical detection rates do fall as conditions become less common; thus, it is incumbent upon laboratories offering large ECS panels to demonstrate the clinical detection rate of screened conditions.

Many factors influence ECS panel content, and our study has been purposefully limited to an evaluation of clinically useful metrics. Other factors that could affect panel size include the clinical utility of screened diseases and the economic feasibility of testing a large panel. However, we have recently demonstrated that disease severity, not rarity, is a driver of ECS clinical utility,^21^ and that the high-throughput of NGS testing enables cost-effective carrier screening of the 176-condition panel explored here.^22^ Additional limitations include that we did not explicitly evaluate the increased clinical burden associated with screening rare conditions including partner testing, genetic counseling, and patient anxiety.^2^ Further, although our estimation of clinical detection rate attempted to account for unobserved pathogenic variants, we made assumptions about the number and frequency of unknown variants. Future research is needed to refine these estimates, yet we demonstrated that our conclusions are robust to a fourfold higher abundance of unknown variants.

In summary, we have shown the first data-driven evaluation in a large patient cohort of the impact on carrier and at-risk couple detection of ECS panel condition inclusion criteria recommended by medical societies. While guidelines are needed to ensure high clinical utility of ECS panels, we showed that the 1-in-100 carrier rate threshold is not supported by data and limits detection of at-risk couples without minimizing residual risk. Instead, we propose that the clinical detection rate of a severe condition may be a better determinant of its suitability for screening than its carrier rate alone.

## Supplementary information

Supplementary Material

Supplementary TableS1

Supplementary TableS2

Supplementary TableS3

Supplementary TableS4
