# Supplementary material for: A data-driven evaluation of the size and content of expanded carrier screening panels
Source: Genet Med. 2019 Feb 28;21(9):1931–9. doi: 10.1038/s41436-019-0466-5 (PMC6752311; doi:10.1038/s41436-019-0466-5)

A Data-Driven Evaluation of the Size and Content of Expanded Carrier Screening Panels Supplementary Information

Rotem Ben-Shachar PhD[1], Ashley Svenson MS CGC[1], James D. Goldberg MD[1], Dale Muzzey PhD[1]

[1] Myriad Women's Health, Inc. (Counsyl, Inc., has transitioned into Myriad Women's Health, Inc.), 180 Kimball Way, South San Francisco, CA, USA

Supplementary Text S1: ACOG classification criteria

It was necessary to make reasonable assumptions regarding the first six criteria definitions because most syndromes can have variable presentations. A condition was determined to "have a well defined phenotype" if a consistent and predictable natural history has been observed across at least 50% of reported cases. Similarly, a condition was considered to "have a detrimental effect on quality of life" if at least 50% of individuals reported to be affected experience severe disease characteristics, defined as Tier 1/Tier 2/Tier 3 disease characteristic classifications in Lazarin et al.[1] Likewise, a condition was determined to "cause cognitive or physical impairment" or "require surgical or medical intervention" if this criteria applied to at least 50% of affected individuals reported in the literature. Finally, we determined that a syndrome has "early onset in life" if at least 50% of individuals reported to be affected have a diagnosis before age 12 years.

Supplementary Text S2: Details of panel analysis computations

Carrier frequencies:

We computed ethnicity-specific allele frequencies for each pathogenic variant observed in our patient cohort. The ethnicity-specific carrier rate for each condition was defined as the proportion of patients of a given ethnicity in our cohort who had at least one pathogenic variant associated with that condition. We defined "panel carrier rate" as the proportion of patients in the cohort who were carriers of at least one condition on the ECS panel.

At-risk couple rates:

At-risk couple rates are computed from two data sources: (1) Expected at-risk couple rates were computed from carrier frequencies, assuming that all male and female patients in a given

ethnicity have the same carrier rate. For AR conditions, the at-risk couple rate is the probability that both members of the couple are carriers (i.e., the condition's carrier-rate squared). For X-linked conditions, the at-risk couple rate is defined as the condition carrier rate. At-risk couple rates were computed for each self-reported ethnicity from ethnicity-specific carrier rates, and then weighted based on U.S.-census frequencies. (2) An empirical observed at-risk couple rate was computed from the cohort in which both partners in a couple were screened. We define the panel at-risk couple rate as the probability that a couple is at risk for any condition on the ECS panel. By making the simplifying assumption that a couple cannot be at risk for multiple conditions, the panel at-risk couple rate is the sum of at-risk couple rates for individual conditions on the panel.

Special cases:

Several conditions on the ECS panel have complicated inheritance. These conditions include alpha thalassemia, fragile X syndrome, spinal muscular atrophy, and 21-OH congenital adrenal hyperplasia. Calculation of carrier frequencies and/or at-risk couple rates for these special cases are provided below:

Spinal muscular atrophy (SMA):

As described previously,[2] our SMA quantitative PCR assay yields total *SMN1* copy number without phase. We used maximum likelihood estimation from observed copy number counts to estimate population-specific alleles frequencies for: 0 *SMN1* copies, 1, or 2+ functional *SMN1* copies, and 1 disabled *SMN1* copy. We define the carrier rate of SMA as the probability of having at least 1 functional copy of *SMN1*. SMA at-risk couple rate is calculated the same as for any AR condition.

Fragile X syndrome:

We define a carrier of fragile X syndrome as an individual with one or two pre-mutations with 55 of more CGG repeats.[2] We assume the risk of developing fragile X syndrome is the sum of the product of the probability of carrying one or two pre-mutations and the probability of the allele expanding to a full mutation, as described previously.[2]

Alpha thalassemia:

Alpha thalassemia displays complex inheritance, where the severity of the condition depends on the number of copies of the *HBA* genes (*HBA1* and *HBA2*). An individual is affected with Hb H if they have 1 functional copy of either of the *HBA* genes. An individual is affected with the more severe Hb Barts if they have no functional copies of either gene. As done previously, we count the number of chromosomes with 0, 1, 2, and 3 or greater functional *HBA* genes.[2] We define an individual as a carrier of Hb H if he/she has one chromosome with 0 or 1 copies and at least two copies in total. We define an individual as a carrier of Hb Barts if he/she has one chromosome with 0 copies and at least one copy in total. We define the ethnicity-specific carrier rate of alpha thalassemia as the sum of the carrier rate of Hb H and the carrier rate of Hb Barts. The at-risk couple rate for alpha thalassemia is defined as the sum of the at-risk couple rate for Hb H and the at-risk couple rate for Hb Barts. The at-risk couple rate for Hb Barts is straightforward, and is computed as is any AR condition. The at-risk couple rate for Hb H is determined by the probability that one parent carries 0 copies on one chromosome and the other parent carries 1 copy on one chromosome.

21-Hydroxylase-deficient Congenital Adrenal Hyperplasia (CAH):

CAH is caused by pathogenic variants in the *CYP21A2* gene. Pathogenic variants often come from recombination of *CYP21A2* with its pseudogene *CYP21AP*. CAH can present in two forms: classical (severe) and non-classical (moderate). For simplicity, we only consider classical-CAH here. We define three alleles types based on *CYP21A2* copy number: classical CAH alleles, nonclassical CAH alleles, and wildtype CAH alleles. We define the probability that an individual is a CAH carrier as the probability than an individual carries any classical CAH allele. CAH at-risk couple rate is calculated the same as for any AR condition.

Supplementary Text S3: Estimating clinical detection rates:

Details of clinical detection rate estimation:

As discussed in Methods, we assume that a minority of pathogenic variants are unobserved in our dataset, that these variants are rare, and that no case reports exist to correctly determine their pathogenicity. To estimate the percentage of unobserved pathogenic variants, we make two conservative assumptions. First, we assume that the number of unobserved variants is equal to half the total number of observed variants, rounded to the highest integer. Second, we assume that the frequency of each unobserved variant is equal to the frequency of the least common observed pathogenic variants. For example, if a condition has 100 observed pathogenic variants and the least common variant has a frequency of 10^{-5} in the population, we would assume that there are 50 unobserved pathogenic variants that each have a frequency of 10^{-5}. If a condition has only one observed pathogenic variant, we would assume that there is one unobserved pathogenic variant with the same variant frequency as the observed pathogenic variant, reducing the estimated clinical detection rate by 50%. The estimated percentage of unobserved pathogenic variants for each condition is shown in Figure S2.

We did not want to assume that all expected cases of a condition are reported. To develop a data-driven proxy for the expected percentage of cases that will be reported in the literature, we utilized an internal literature database of reported cases per condition. Figure S3A shows the total number of cases of unrelated patients per condition compared to the U.S.-weighted carrier rate. As expected, the number of reported cases is higher for conditions with high carrier rates compared to conditions with low carrier rates. Figure S3B shows the ratio of the total number of cases of unrelated patients per condition and the expected number of cases per conditions, defined as the product of U.S-weighted prevalence of each condition and the world population estimate. This ratio is close to 1 for conditions with low carrier rates, signifying that for rare conditions, most cases are reported. Alternatively, this ratio is low for conditions with high carrier rates, signifying that for prevalent conditions, only a small fraction of cases are reported in the literature. To more accurately simulate the expected number of reported cases per condition, we multiply the simulated number of cases for each pathogenic variant for each condition by this ratio of total number of cases of unrelated patients per condition compared to the U.S.-weighted carrier rate.

In summary, for each condition, we counted the number of observed pathogenic variants, with ethnicity-specific counts weighted by U.S.-census frequencies. We then normalized the weighted counts to yield relative variant frequencies (i.e., normalized frequencies for each condition summed to one). Next, we computed the expected number of reported symptomatic cases of each condition worldwide, based on the product of the world population (7.6 billion), the expected

fraction of cases that will be reported, and the worldwide condition prevalence (25% the U.S.-weighted at-risk couple rate was used as a proxy for worldwide prevalence).

Conditions excluded from clinical detection rate estimation:

We excluded X-linked severe combined immunodeficiency (*IL2RG*) and X-linked ornithine transcarbamylase deficiency (*OTC*) from the clinical detection rate estimation as we have not observed any carriers of this condition during this study period in U.S.-specific ethnicities. Analysis of other X-linked conditions shows that X-linked conditions typically have few observed pathogenic variants. Additionally, our internal database of reported cases indicate that there are several hundred reported cases of patients affected with *IL2RG* or *OTC*. Taken together, these results indicate that the pathogenicity of unobserved variants for these conditions can be determined based on case reports, ensuring high clinical detection rates.

Sensitivity of clinical detection rate estimation:

To assess how sensitive our clinical detection rate estimates were to the assumption that three or more reported cases are needed to interpret the pathogenicity of observed variants, we estimated clinical detection rate assuming that one, three and four reported cases are needed to interpret the pathogenicity of observed variants (Supplementary Figures S4-S6). Estimated clinical detection rate decreases as additional reported cases are required to interpret pathogenicity. However, clinical detection rate estimates are relatively robust to this number. The average estimated clinical detection rate across all conditions drops from 97.6% to 96.1% when we assume one reported case as opposed to four reported cases are needed to determine variant pathogenicity.

We further estimated the impact on clinical detection rate when we increase the assumed frequency of unobserved variants. If the the number of unobserved variants is increased 4x higher than initially assumed, clinical-detection rates change to a small extent (>90% for the majority of conditions (Figure S7), suggesting our conclusions are robust to the rate of unknown variants.

References

1. Lazarin, G. A. *et al.* Systematic Classification of Disease Severity for Evaluation of Expanded Carrier Screening Panels. *PLoS One* **9,** e114391 (2014).

2. Haque, I. S. *et al.* Modeled Fetal Risk of Genetic Diseases Identified by Expanded Carrier Screening. *JAMA* **316,** 734–742 (2016).

SUPPLEMENTARY FIGURES

<u>FIGURE S1:</u> Distribution of observed at-risk couples. Two cohorts of at-risk couples are shown: all observed at-risk couples (light colors, cohort 1) and observed at-risk couples with no family or personal history or consanguinity (dark colors, cohort 2, see Methods). Conditions in pink have a U.S.-weighted carrier rate >1-in-100 for AR conditions and >1-in-10,000 for X-linked conditions; conditions in yellow have U.S.-weighted carrier rates below these thresholds. The distribution of conditions is similar for both cohorts. In Cohort 1, 51 out of 69 conditions have carrier rates below the 1-in-100 threshold. In Cohort 2, 34 out of 52 conditions have carrier rates below the 1-in-100 threshold.

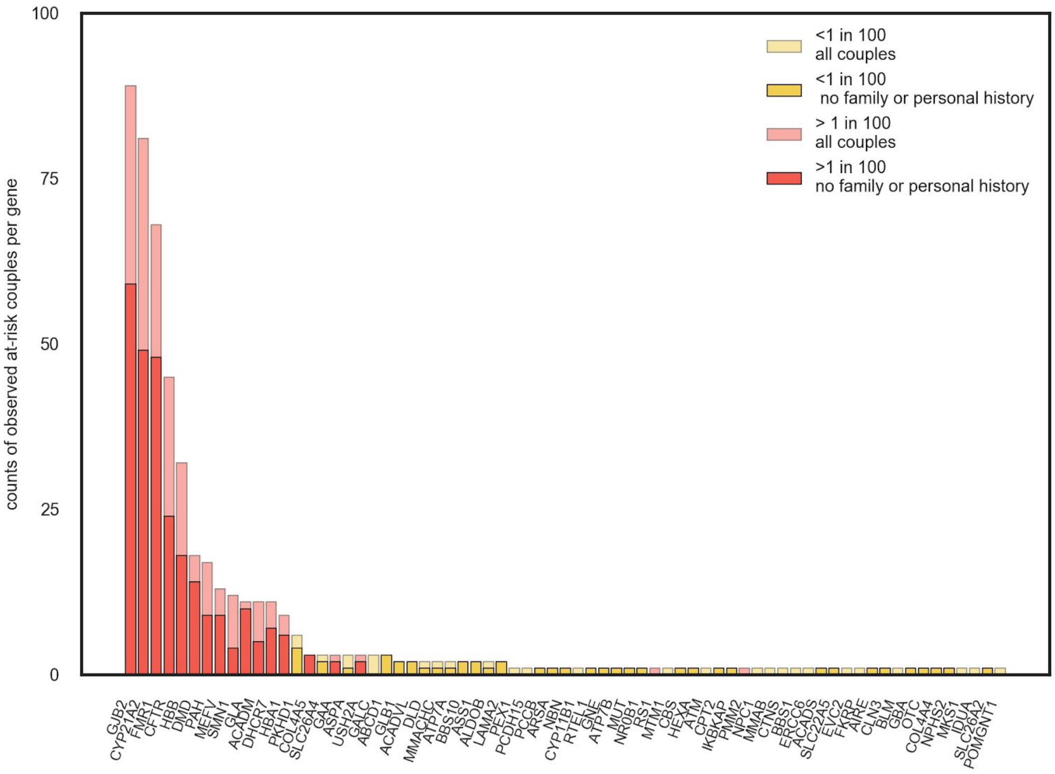

FIGURE S2: The percentage of estimated unobserved variants for each condition. These values are used to estimate clinical detection rate. Each dot denotes a condition on the 176-condition panel.

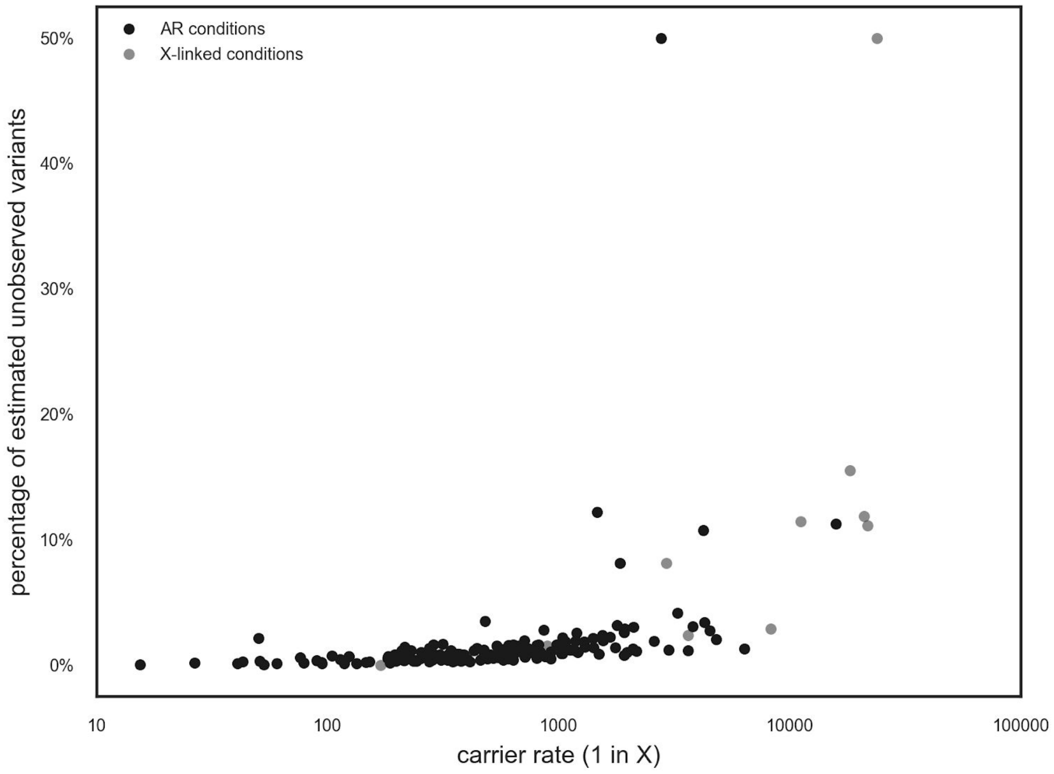

<u>FIGURE S3:</u> (A) The relationship between carrier rate and the number of cases from unrelated patients, a proxy for reported cases, for each condition. (B) The relationship between carrier rate and the ratio of number of cases from unrelated patients (shown in A) to expected cases worldwide based on the methodology described above. This ratio is a proxy for the expected number of cases per condition that we expected to be reported for each condition.

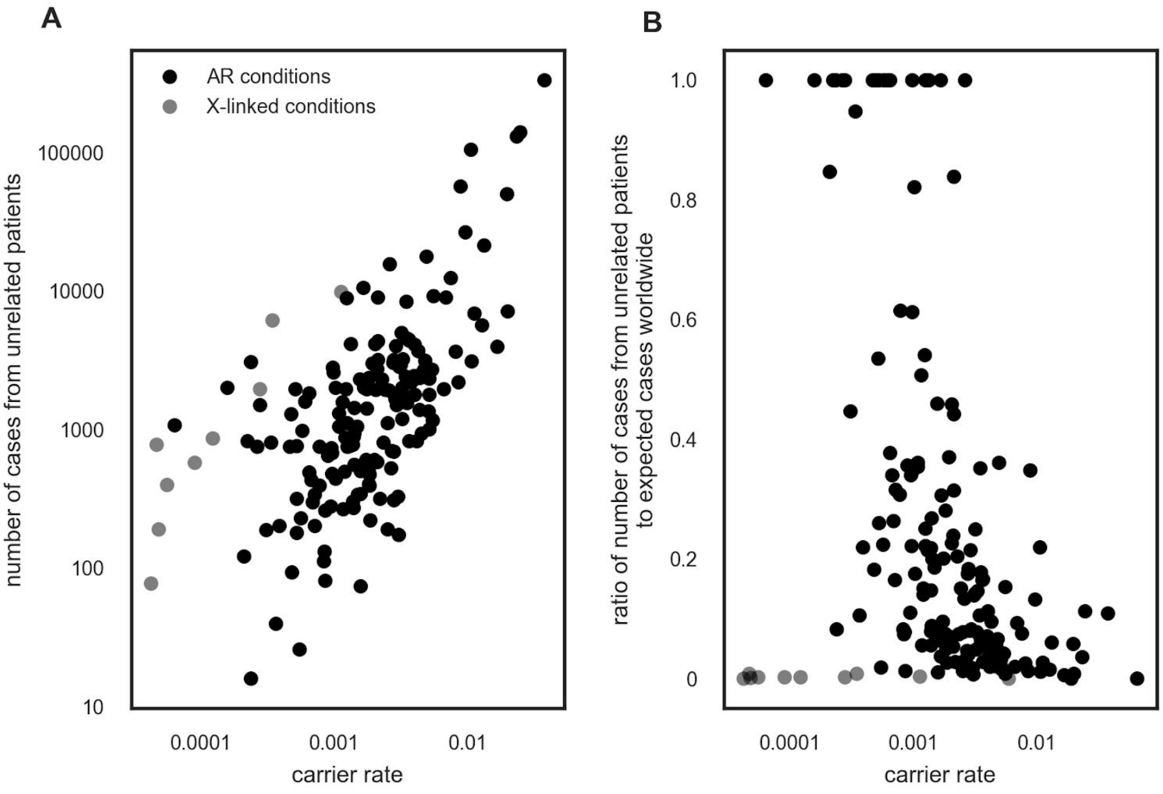

FIGURE S4: U.S.-weighted carrier rate and estimated clinical detection rate for each condition are shown for each condition when we assume one reported case is needed to determine pathogenicity for each variant. Dots show median estimated clinical detection rates and lines show corresponding 95% confidence intervals. (A) AR conditions. (B) X-linked conditions.

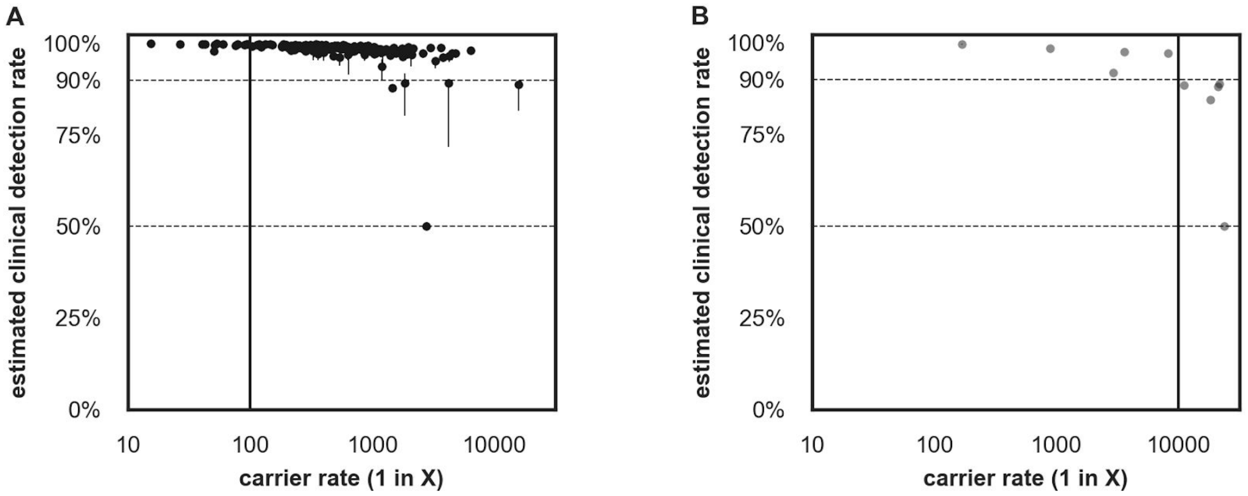

FIGURE S5: U.S.-weighted carrier rate and estimated clinical detection rates for each condition are shown for each condition when we assume two reported cases are needed to determine pathogenicity for each variant. Dots show median estimated clinical detection rate and lines show corresponding 95% confidence intervals. (A) AR conditions. (B) X-linked conditions.

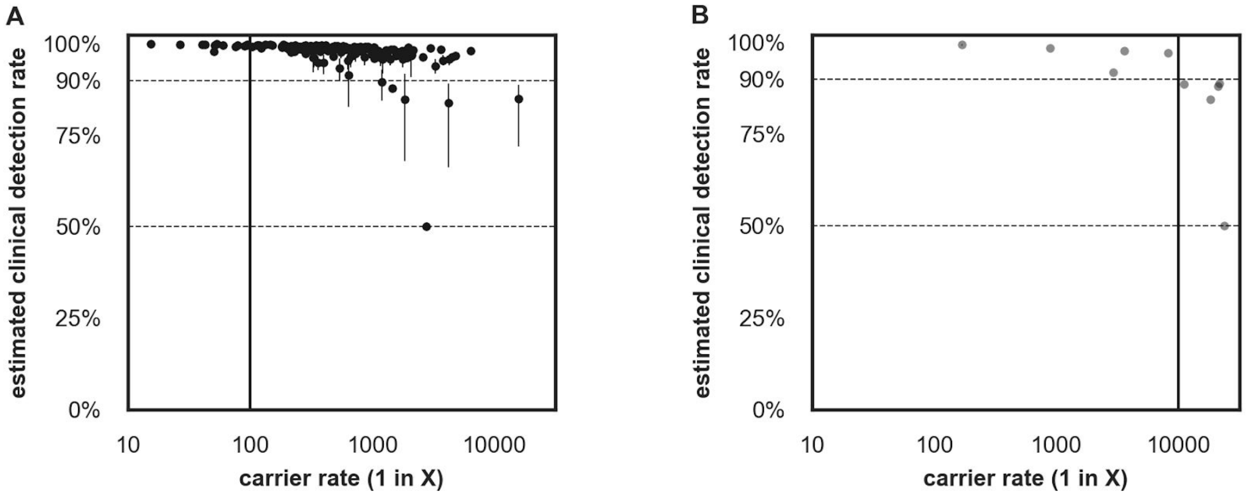

FIGURE S6: U.S.-weighted carrier rate and estimated clinical detection rate for each condition are shown for each condition when we assume four reported cases are needed to determine pathogenicity for each variant. Dots show median estimated clinical detection rates and lines show corresponding 95% confidence intervals. (A) AR conditions. (B) X-linked conditions.

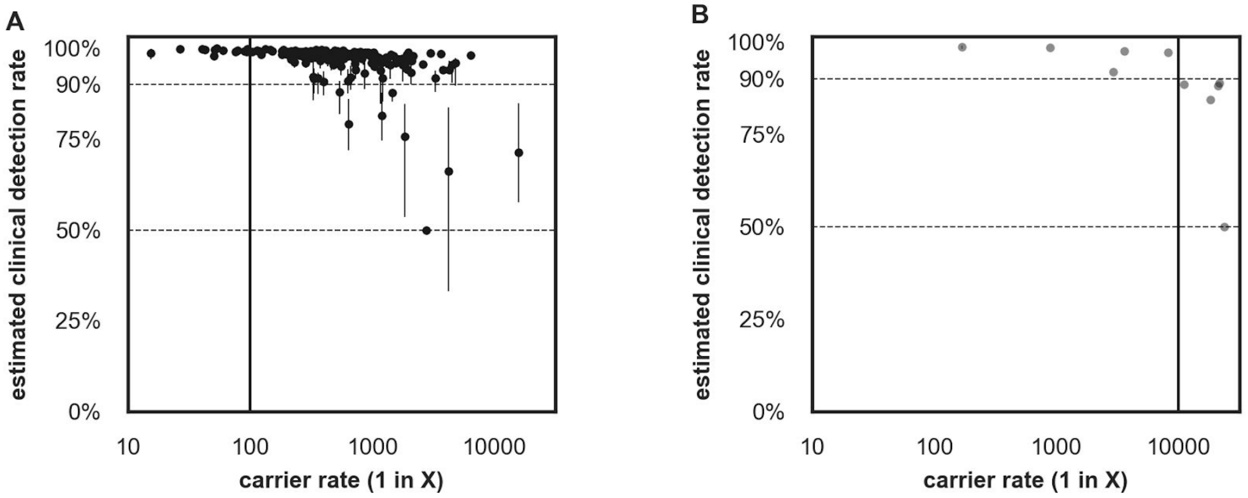

FIGURE S7: U.S.-weighted carrier rate and estimated clinical detection rate for each condition are shown for each condition when we assume three reported cases are needed to determine pathogenicity for each variant and that the number of unobserved variants is equal to two-times the total number of observed variants, rounded to the highest integer (i.e., 4x more unobserved variants than assumed in Figure 5 and Figure S4-S6). Dots show median estimated clinical detection rates and lines show corresponding 95% confidence intervals. (A) AR conditions. (B) X-linked conditions.

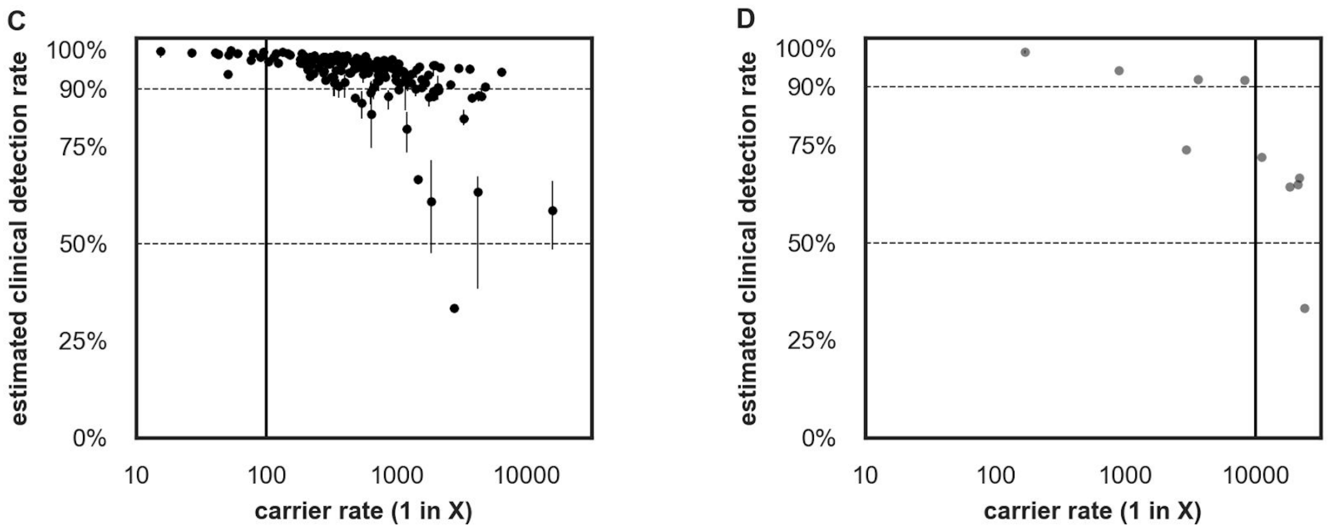

Supplement: Supplementary file 1 — Supplementary Material [file 41436_2019_466_MOESM1_ESM.pdf]
