# Supplementary figures and images for: A data-driven evaluation of the size and content of expanded carrier screening panels
Source: Genet Med. 2019 Feb 28;21(9):1931–9. doi: 10.1038/s41436-019-0466-5 (PMC6752311; doi:10.1038/s41436-019-0466-5)

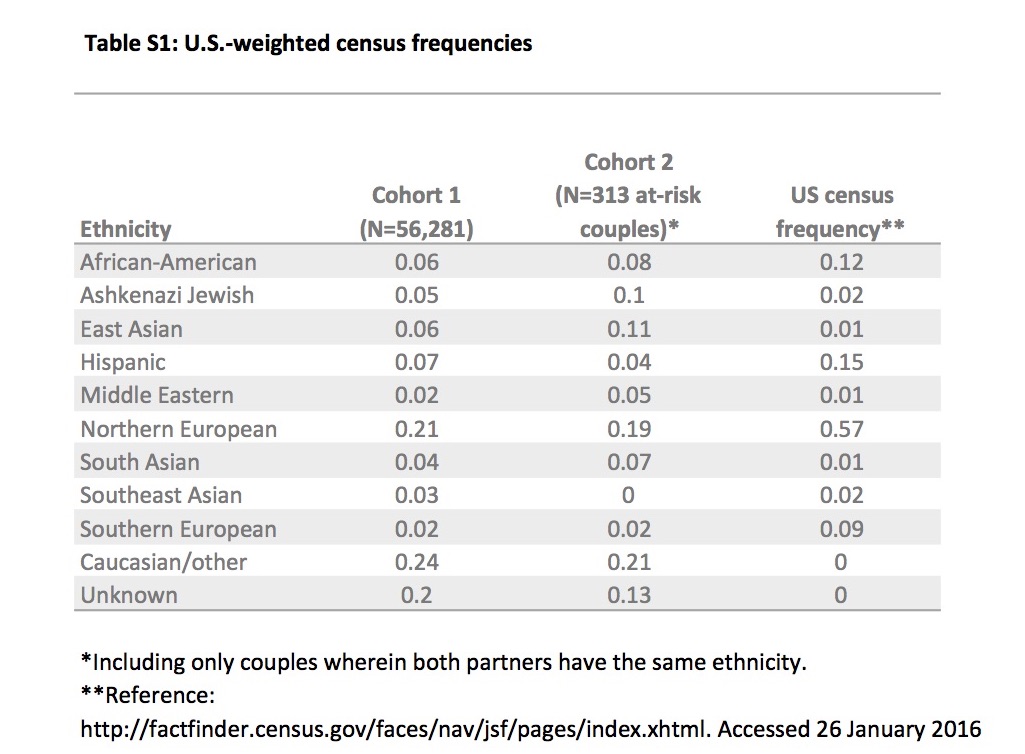

Supplement: Supplementary file 2 — Supplementary TableS1 [file 41436_2019_466_MOESM2_ESM.jpg]
