# Supplementary material for: A data-driven evaluation of the size and content of expanded carrier screening panels
Source: Genet Med. 2019 Feb 28;21(9):1931–9. doi: 10.1038/s41436-019-0466-5 (PMC6752311; doi:10.1038/s41436-019-0466-5)
Supplement: Supplementary file 3 — Supplementary TableS2 [file 41436_2019_466_MOESM3_ESM.pdf]

Table S2: Six ACOG criteria classification of a 176-condition panel

Disease name	Has a well-defined phenotype	Detrimental effect on quality of life in most affected individuals	May cause cognitive or physical impairment	Typically requires medical or surgical intervention	Often has childhood onset	Prenatal diagnosis is available
11-beta-hydroxylase-deficient congenital adrenal hyperplasia	X	X	X	X	X	X
congenital adrenal hyperplasia	X	X	X	X	X	X
6-pyruvoyl-tetrahydropterin synthase deficiency	X	X	X	X	X	X
ABCC8-related hyperinsulinism	X	X	X	X	X	X
AMT-related glycine encephalopathy	X	X	X	X	X	X
ARSACS	X	X	X	X	X	X
ATP7A-related disorders	X	X	X	X	X	X
Alstrom syndrome	X	X	X	X	X	X
Andermann syndrome	X	X	X	X	X	X
Bardet-Biedl syndrome, BBS1-related	X	X	X	X	X	X
Bardet-Biedl syndrome, BBS10-related	X	X	X	X	X	X
Bardet-Biedl syndrome, BBS12-related	X	X	X	X	X	X
Bardet-Biedl syndrome, BBS2-related	X	X	X	X	X	X
Bloom syndrome	X	X	X	X	X	X
CLN3-related neuronal ceroid lipofuscinosis	X	X	X	X	X	X
CLN5-related neuronal ceroid lipofuscinosis	X	X	X	X	X	X
CLN6-related neuronal ceroid lipofuscinosis	X	X	X	X	X	X
COL4A3-related Alport syndrome	X	X	X	X	X	X
COL4A4-related Alport syndrome	X	X	X	X	X	X
Canavan disease	X	X	X	X	X	X
Cohen syndrome	X	X	X	X	X	X
Costeff optic atrophy syndrome	X	X	X	X	X	X
D-bifunctional protein deficiency	X	X	X	X	X	X
ERCC6-related disorders	X	X	X	X	X	X
ERCC8-related disorders	X	X	X	X	X	X
EVC-related Ellis-van Creveld syndrome	X	X	X	X	X	X
EVC2-related Ellis-van Creveld syndrome	X	X	X	X	X	X
FKRP-related disorders	X	X	X	X	X	X
FKTN-related disorders	X	X	X	X	X	X
Fabry disease	X	X	X	X	X	X
Fanconi anemia complementation group A	X	X	X	X	X	X
Fanconi anemia type C	X	X	X	X	X	X
GJB2-related DFNB1 nonsyndromic hearing loss and deafness	X	X	X	X	X	X
GLB1-related disorders	X	X	X	X	X	X
GLDC-related glycine encephalopathy	X	X	X	X	X	X
GNPTAB-related disorders	X	X	X	X	X	X
GRACILE syndrome	X	X	X	X	X	X
Gaucher disease	X	X	X	X	X	X
HADHA-related disorders	X	X	X	X	X	X
HMG-CoA lyase deficiency	X	X	X	X	X	X
Hb beta chain-related hemoglobinopathy	X	X	X	X	X	X
Herlitz junctional epidermolysis bullosa, LAMA3-related	X	X	X	X	X	X
Herlitz junctional epidermolysis bullosa, LAMB3-related	X	X	X	X	X	X
Herlitz junctional epidermolysis bullosa, LAMC2-related	X	X	X	X	X	X
Joubert syndrome 2	X	X	X	X	X	X
KCNJ11-related familial hyperinsulinism	X	X	X	X	X	X
Krabbe disease	X	X	X	X	X	X
LAMA2-related muscular dystrophy	X	X	X	X	X	X
Leigh syndrome, French-Canadian type	X	X	X	X	X	X
MKS1-related disorders	X	X	X	X	X	X
MUT-related methylmalonic acidemia	X	X	X	X	X	X

Table S2: Six ACOG criteria classification of a 176-condition panel

MYO7A-related disorders	X	X	X	X	X	X
NEB-related nemaline myopathy	X	X	X	X	X	X
Niemann-Pick disease type C	X	X	X	X	X	X
Niemann-Pick disease type C2	X	X	X	X	X	X
Niemann-Pick disease, SMPD1-associated	X	X	X	X	X	X
Nijmegen breakage syndrome	X	X	X	X	X	X
Northern epilepsy	X	X	X	X	X	X
PCCA-related propionic acidemia	X	X	X	X	X	X
PCCB-related propionic acidemia	X	X	X	X	X	X
PCDH15-related disorders	X	X	X	X	X	X
PEX1-related Zellweger syndrome spectrum	X	X	X	X	X	X
PKHD1-related autosomal recessive polycystic kidney disease	X	X	X	X	X	X
PPT1-related neuronal ceroid lipofuscinosis	X	X	X	X	X	X
PROP1-related combined pituitary hormone deficiency	X	X	X	X	X	X
Pendred syndrome	X	X	X	X	X	X
Pompe disease	X	X	X	X	X	X
RTEL1-related disorders	X	X	X	X	X	X
Salla disease	X	X	X	X	X	X
Sandhoff disease	X	X	X	X	X	X
Segawa syndrome	X	X	X	X	X	X
Sjogren-Larsson syndrome	X	X	X	X	X	X
Smith-Lemli-Opitz syndrome	X	X	X	X	X	X
TGM1-related autosomal recessive congenital ichthyosis	X	X	X	X	X	X
TPP1-related neuronal ceroid lipofuscinosis	X	X	X	X	X	X
USH1C-related disorders	X	X	X	X	X	X
USH2A-related disorders	X	X	X	X	X	X
Usher syndrome type 3	X	X	X	X	X	X
Wilson disease	X	X	X	X	X	X
X-linked Alport syndrome	X	X	X	X	X	X
X-linked adrenoleukodystrophy	X	X	X	X	X	X
X-linked congenital adrenal hypoplasia	X	X	X	X	X	X
X-linked juvenile retinoschisis	X	X	X	X	X	X
X-linked myotubular myopathy	X	X	X	X	X	X
X-linked severe combined immunodeficiency	X	X	X	X	X	X
adenosine deaminase deficiency	X	X	X	X	X	X
alpha thalassemia	X	X	X	X	X	X
alpha-mannosidosis	X	X	X	X	X	X
alpha-sarcoglycanopathy	X	X	X	X	X	X
argininemia	X	X	X	X	X	X
argininosuccinic aciduria	X	X	X	X	X	X
aspartylglycosaminuria	X	X	X	X	X	X
ataxia with vitamin E deficiency	X	X	X	X	X	X
ataxia-telangiectasia	X	X	X	X	X	X
autosomal recessive osteopetrosis type 1	X	X	X	X	X	X
beta-sarcoglycanopathy	X	X	X	X	X	X
biotinidase deficiency	X	X	X	X	X	X
calpainopathy	X	X	X	X	X	X
carbamoylphosphate synthetase I deficiency	X	X	X	X	X	X
carnitine palmitoyltransferase IA deficiency	X	X	X	X	X	X
carnitine palmitoyltransferase II deficiency	X	X	X	X	X	X
cartilage-hair hypoplasia	X	X	X	X	X	X
cerebrotendinous xanthomatosis	X	X	X	X	X	X
citrullinemia type 1	X	X	X	X	X	X
congenital Finnish nephrosis	X	X	X	X	X	X
congenital disorder of glycosylation type Ia	X	X	X	X	X	X
congenital disorder of glycosylation type Ib	X	X	X	X	X	X
congenital disorder of glycosylation type Ic	X	X	X	X	X	X

Table S2: Six ACOG criteria classification of a 176-condition panel

cystic fibrosis	X	X	X	X	X	X
cystinosis	X	X	X	X	X	X
delta-sarcoglycanopathy	X	X	X	X	X	X
dysferlinopathy	X	X	X	X		X
dystrophinopathy (including Duchenne/Becker muscular dystrop	X	X	X	X	X	X
familial Mediterranean fever	X	X	X	X		X
familial dysautonomia	X	X	X	X	X	X
fragile X syndrome	X	X	X	X	X	X
galactokinase deficiency	X	X	X	X	X	X
galactosemia	X	X	X	X	X	X
gamma-sarcoglycanopathy	X	X	X	X	X	X
glutaric acidemia type 1	X	X	X	X	X	X
glycogen storage disease type III	X	X	X	X	X	X
glycogen storage disease type Ia	X	X	X	X	X	X
glycogen storage disease type Ib	X	X	X	X	X	X
hereditary fructose intolerance	X	X	X	X	X	X
hexosaminidase A deficiency	X	X	X	X	X	X
holocarboxylase synthetase deficiency	X	X	X	X	X	X
homocystinuria caused by cystathionine beta-synthase deficienc	X	X	X	X	X	X
hydrolethalus syndrome	X	X	X	X	X	X
hypophosphatasia, autosomal recessive	X	X	X	X	X	X
inclusion body myopathy 2	X	X	X	X	X	X
isovaleric acidemia	X	X	X	X	X	X
lipoamide dehydrogenase deficiency	X	X	X	X	X	X
lipoid congenital adrenal hyperplasia	X	X	X	X	X	X
lysosomal acid lipase deficiency	X	X	X	X		X
maple syrup urine disease type 1B	X	X	X	X	X	X
maple syrup urine disease type II	X	X	X	X	X	X
maple syrup urine disease type Ia	X	X	X	X	X	X
medium chain acyl-CoA dehydrogenase deficiency	X	X	X	X	X	X
megalencephalic leukoencephalopathy with subcortical cysts	X	X	X	X	X	X
metachromatic leukodystrophy	X	X	X	X	X	X
methylmalonic acidemia, cblA type	X	X	X	X	X	X
methylmalonic acidemia, cblB type	X	X	X	X	X	X
methylmalonic aciduria and homocystinuria, cblC type	X	X	X	X	X	X
mucolipidosis III gamma	X	X	X	X	X	X
mucolipidosis IV	X	X	X	X	X	X
mucopolysaccharidosis type I	X	X	X	X	X	X
mucopolysaccharidosis type II	X	X	X	X	X	X
mucopolysaccharidosis type IIIA	X	X	X	X	X	X
mucopolysaccharidosis type IIIB	X	X	X	X	X	X
mucopolysaccharidosis type IIIC	X	X	X	X	X	X
muscle-eye-brain disease	X	X	X	X	X	X
nephrotic syndrome, NPHS2-related	X	X	X	X	X	X
ornithine transcarbamylase deficiency	X	X	X	X	X	X
peroxisome biogenesis disorder type 3	X	X	X	X	X	X
peroxisome biogenesis disorder type 4	X	X	X	X	X	X
peroxisome biogenesis disorder type 5	X	X	X	X	X	X
peroxisome biogenesis disorder type 6	X	X	X	X	X	X
phenylalanine hydroxylase deficiency	X	X	X	X	X	X
polyglandular autoimmune syndrome type 1	X	X	X	X	X	X
primary carnitine deficiency	X	X	X	X	X	X
primary hyperoxaluria type 1	X	X	X	X	X	X
primary hyperoxaluria type 2	X	X	X	X	X	X
primary hyperoxaluria type 3	X	X	X	X	X	X
pycnodysostosis	X	X	X	X	X	X
pyruvate carboxylase deficiency	X	X	X	X	X	X

Table S2: Six ACOG criteria classification of a 176-condition panel

rhizomelic chondrodysplasia punctata type 1	X	X	X	X	X	X
short chain acyl-CoA dehydrogenase deficiency	X		X			X
spastic paraplegia type 15	X	X	X	X	X	X
spinal muscular atrophy	X	X	X	X	X	X
spondylothoracic dysostosis	X	X	X	X	X	X
sulfate transporter-related osteochondrodysplasia	X	X	X	X	X	X
tyrosinemia type I	X	X	X	X	X	X
tyrosinemia type II	X	X	X	X	X	X
very long chain acyl-CoA dehydrogenase deficiency	X	X	X	X	X	X
xeroderma pigmentosum group A	X	X	X	X	X	X
xeroderma pigmentosum group C	X	X	X	X	X	X
