# Supplementary material for: A data-driven evaluation of the size and content of expanded carrier screening panels
Source: Genet Med. 2019 Feb 28;21(9):1931–9. doi: 10.1038/s41436-019-0466-5 (PMC6752311; doi:10.1038/s41436-019-0466-5)
Supplement: Supplementary file 4 — Supplementary TableS3 [file 41436_2019_466_MOESM4_ESM.pdf]

Table S3: 1-in-100 carrier frequency criteria classifications of 176-condition panel

Disease name	Any ethnicity (relaxed X-linked criteria)*	All ethnicities (relaxed X-linked criteria)*	U.S.-population (relaxed X-linked criteria)*	Any ethnicity (stringent X-linked criteria)* in at least one ethnicity	All ethnicities (stringent X-linked criteria)*	U.S.-population (stringent X-linked criteria)*
11-beta-hydroxylase-deficient congenital adrenal hyperplasia						
congenital adrenal hyperplasia	X	X	X	X	X	X
6-pyruvoyl-tetrahydropterin synthase deficiency						
ABCC8-related hyperinsulinism						
AMT-related glycine encephalopathy						
ARSACS						
ATP7A-related disorders	X	X				
Alstrom syndrome						
Andermann syndrome						
Bardet-Biedl syndrome, BBS1-related						
Bardet-Biedl syndrome, BBS10-related						
Bardet-Biedl syndrome, BBS12-related						
Bardet-Biedl syndrome, BBS2-related						
Bloom syndrome						
CLN3-related neuronal ceroid lipofuscinosis						
CLN5-related neuronal ceroid lipofuscinosis						
CLN6-related neuronal ceroid lipofuscinosis						
COL4A3-related Alport syndrome						
COL4A4-related Alport syndrome						
Canavan disease	X			X		
Cohen syndrome						
Costeff optic atrophy syndrome						
D-bifunctional protein deficiency						
ERCC6-related disorders						
ERCC8-related disorders						
EVC-related Ellis-van Creveld syndrome						
EVC2-related Ellis-van Creveld syndrome						
FKRP-related disorders						
FKTN-related disorders	X			X		
Fabry disease	X	X	X			
Fanconi anemia complementation group A						
Fanconi anemia type C						
GJB2-related DFNB1 nonsyndromic hearing loss and deafness	X		X	X		X
GLB1-related disorders						
GLDC-related glycine encephalopathy						
GNPTAB-related disorders						
GRACILE syndrome						
Gaucher disease	X			X		
HADHA-related disorders						
HMG-CoA lyase deficiency						
Hb beta chain-related hemoglobinopathy	X		X	X		X
Herlitz junctional epidermolysis bullosa, LAMA3-related						
Herlitz junctional epidermolysis bullosa, LAMB3-related						
Herlitz junctional epidermolysis bullosa, LAMC2-related						
Joubert syndrome 2						
KCNJ11-related familial hyperinsulinism						
Krabbe disease	X			X		
LAMA2-related muscular dystrophy						
Leigh syndrome, French-Canadian type						
MKS1-related disorders						
MUT-related methylmalonic acidemia						
MYO7A-related disorders						
NEB-related nemaline myopathy	X			X		
Niemann-Pick disease type C						
Niemann-Pick disease type C2						
Niemann-Pick disease, SMPD1-associated						
Nijmegen breakage syndrome						
Northern epilepsy						
PCCA-related propionic acidemia						
PCCB-related propionic acidemia						
PCDH15-related disorders						
PEX1-related Zellweger syndrome spectrum						
PKHD1-related autosomal recessive polycystic kidney disease	X			X		
PPT1-related neuronal ceroid lipofuscinosis						
PROP1-related combined pituitary hormone deficiency						
Pendred syndrome	X			X		
Pompe disease	X		X	X		X
RTEL1-related disorders						
Salla disease						
Sandhoff disease						
Segawa syndrome						
Sjogren-Larsson syndrome						
Smith-Lemli-Opitz syndrome	X		X	X		X
TGM1-related autosomal recessive congenital ichthyosis						
TPP1-related neuronal ceroid lipofuscinosis						
USH1C-related disorders						
USH2A-related disorders	X		X	X		X
Usher syndrome type 3						
Wilson disease	X			X		
X-linked Alport syndrome	X	X	X			
X-linked adrenoleukodystrophy	X	X				
X-linked congenital adrenal hypoplasia	X	X				
X-linked juvenile retinoschisis	X		X			
X-linked myotubular myopathy	X	X				
X-linked severe combined immunodeficiency						
adenosine deaminase deficiency						
alpha thalassemia	X	X	X	X	X	X
alpha-mannosidosis						

*Relaxed X-linked criteria: 1-in-10,000 carrier rate threshold for X-linked conditions
Stringent X-linked criteria: 1-in-100 carrier rate threshold for X-linked conditions

Table S3: 1-in-100 carrier frequency criteria classifications of 176-condition panel

alpha-sarcoglycanopathy						
argininemia						
argininosuccinic aciduria						
aspartylglycosaminuria						
ataxia with vitamin E deficiency						
ataxia-telangiectasia						
autosomal recessive osteopetrosis type 1						
beta-sarcoglycanopathy						
biotinidase deficiency						
calpainopathy						
carbamoylphosphate synthetase I deficiency						
carnitine palmitoyltransferase IA deficiency						
carnitine palmitoyltransferase II deficiency	X			X		
cartilage-hair hypoplasia						
cerebrotendinous xanthomatosis						
citrullinemia type 1						
congenital Finnish nephrosis						
congenital disorder of glycosylation type Ia	X		X	X		X
congenital disorder of glycosylation type Ib						
congenital disorder of glycosylation type Ic						
cystic fibrosis	X		X	X		X
cystinosis						
delta-sarcoglycanopathy						
dysferlinopathy						
dystrophinopathy (including Duchenne/Becker muscular dystrophy)	X	X	X			
familial Mediterranean fever	X		X	X		X
familial dysautonomia	X			X		
fragile X syndrome	X	X	X			
galactokinase deficiency						
galactosemia						
gamma-sarcoglycanopathy						
glutaric acidemia type 1						
glycogen storage disease type III						
glycogen storage disease type Ia						
glycogen storage disease type Ib						
hereditary fructose intolerance	X			X		
hexosaminidase A deficiency	X			X		
holocarboxylase synthetase deficiency						
homocystinuria caused by cystathionine beta-synthase deficiency						
hydrolethalus syndrome						
hypophosphatasia, autosomal recessive						
inclusion body myopathy 2						
isovaleric acidemia						
lipoamide dehydrogenase deficiency	X			X		
lipoid congenital adrenal hyperplasia						
lysosomal acid lipase deficiency						
maple syrup urine disease type 1B						
maple syrup urine disease type II						
maple syrup urine disease type Ia						
medium chain acyl-CoA dehydrogenase deficiency	X		X	X		X
megalencephalic leukoencephalopathy with subcortical cysts						
metachromatic leukodystrophy						
methylmalonic acidemia, cblA type						
methylmalonic acidemia, cblB type						
methylmalonic aciduria and homocystinuria, cblC type						
mucolipidosis III gamma						
mucolipidosis IV						
mucopolysaccharidosis type I						
mucopolysaccharidosis type II	X	X				
mucopolysaccharidosis type IIIA						
mucopolysaccharidosis type IIIB						
mucopolysaccharidosis type IIIC						
muscle-eye-brain disease						
nephrotic syndrome, NPHS2-related						
ornithine transcarbamylase deficiency	X	X				
peroxisome biogenesis disorder type 3						
peroxisome biogenesis disorder type 4						
peroxisome biogenesis disorder type 5						
peroxisome biogenesis disorder type 6						
phenylalanine hydroxylase deficiency	X		X	X		X
polyglandular autoimmune syndrome type 1						
primary carnitine deficiency	X			X		
primary hyperoxaluria type 1						
primary hyperoxaluria type 2						
primary hyperoxaluria type 3	X			X		
pycnodysostosis						
pyruvate carboxylase deficiency						
rhizomelic chondrodysplasia punctata type 1						
short chain acyl-CoA dehydrogenase deficiency	X			X		
spastic paraplegia type 15						
spinal muscular atrophy	X	X	X	X	X	X
spondylothoracic dysostosis						
sulfate transporter-related osteochondrodysplasia						
tyrosinemia type I						
tyrosinemia type II						
very long chain acyl-CoA dehydrogenase deficiency						
xeroderma pigmentosum group A						
xeroderma pigmentosum group C						

*Relaxed X-linked criteria: 1-in-10,000 carrier rate threshold for X-linked conditions
Stringent X-linked criteria: 1-in-100 carrier rate threshold for X-linked conditions
