# Supplementary material for: A data-driven evaluation of the size and content of expanded carrier screening panels
Source: Genet Med. 2019 Feb 28;21(9):1931–9. doi: 10.1038/s41436-019-0466-5 (PMC6752311; doi:10.1038/s41436-019-0466-5)
Supplement: Supplementary file 5 — Supplementary TableS4 [file 41436_2019_466_MOESM5_ESM.pdf]

Table S4: Clinical detection rate estimates when three reported cases are assumed to determine pathogenicity

Disease name	U.S. weighted carrier rate	2.5% percentile estimated clinical detection rate	Median estimated clinical detection rate	97.5% percentiile estimated clinical detection rate	Inheritance pattern
medium chain acyl-CoA dehydrogenase deficiency	0.012698476	0.993761828	0.995486917	0.996545552	AR
Fanconi anemia type C	0.001797167	0.983294097	0.985921992	0.988737594	AR
cartilage-hair hypoplasia	0.004522336	0.970381469	0.979765632	0.988033518	AR
GNPTAB-related disorders	0.002686878	0.958074228	0.971918174	0.978994907	AR
Niemann-Pick disease type C2	0.000334853	0.981525435	0.987683623	0.987683623	AR
delta-sarcoglycanopathy	0.00015691	0.980765837	0.980765837	0.980765837	AR
hydrolethalus syndrome	0.000361705	0.5	0.5	0.5	AR
mucolipidosis IV	0.000844505	0.945104183	0.973398089	0.975795081	AR
RTEL1-related disorders	0.001081846	0.982088737	0.984175486	0.987181398	AR
FKRP-related disorders	0.002874647	0.99498661	0.99621081	0.997220046	AR
rhizomelic chondrodysplasia punctata type 1	0.002178531	0.954908905	0.981189977	0.992283068	AR
spastic paraplegia type 15	0.000963224	0.948672613	0.953338739	0.960038818	AR
alpha-sarcoglycanopathy	0.002033247	0.993637005	0.993637005	0.995126216	AR
alpha-mannosidosis	0.001805763	0.943024115	0.966446795	0.982281565	AR
congenital Finnish nephrosis	0.003445259	0.98899908	0.990384017	0.991513576	AR
PEX1-related Zellweger syndrome spectrum	0.00393235	0.979840799	0.984343276	0.986288685	AR
glycogen storage disease type III	0.002926179	0.971335404	0.97996564	0.98164398	AR
homocystinuria caused by cystathionine beta-synthase deficiency	0.006576505	0.989550457	0.994527393	0.996211661	AR
sulfate transporter-related osteochondrodysplasia	0.003633881	0.986073896	0.989657775	0.993241654	AR
ERCC6-related disorders	0.001851924	0.860044191	0.902398955	0.934407148	AR
X-linked adrenoleukodystrophy	5.50E-05	0.844660194	0.844660194	0.844660194	X-linked
mucopolysaccharidosis type II	8.96E-05	0.885553471	0.885553471	0.885553471	X-linked
argininemia	0.000560756	0.927005326	0.95377004	0.958717457	AR
mucopolysaccharidosis type I	0.005102581	0.98325047	0.987935933	0.990110469	AR
Niemann-Pick disease type C	0.00396546	0.98999163	0.991100818	0.992302439	AR
gamma-sarcoglycanopathy	0.001564472	0.970512772	0.988374129	0.989653203	AR
MUT-related methylmalonic acidemia	0.002265689	0.975418735	0.979157771	0.98257963	AR
galactokinase deficiency	0.000387224	0.956919934	0.956919934	0.967205608	AR
Costeff optic atrophy syndrome	6.31E-05	0.647540984	0.784543326	0.887587822	AR
Sjogren-Larsson syndrome	0.001191998	0.968211509	0.981328594	0.9844479	AR
adenosine deaminase deficiency	0.001291887	0.984568637	0.984568637	0.98730646	AR
ERCC8-related disorders	0.000834744	0.791522522	0.857002746	0.904240522	AR
Herlitz junctional epidermolysis bullosa, LAMB3-related	0.002488809	0.979534604	0.988262493	0.992448597	AR
spondylothoracic dysostosis	0.000544056	0.591246581	0.758319055	0.891423934	AR
cystic fibrosis	0.037636752	0.998272784	0.998299772	0.998299772	AR
NEB-related nemaline myopathy	0.004651312	0.967895667	0.972989285	0.975113019	AR
argininosuccinic aciduria	0.004127068	0.982901242	0.986605597	0.991815786	AR
maple syrup urine disease type II	0.001817655	0.966639341	0.978998486	0.987147601	AR
GLB1-related disorders	0.002325725	0.961252466	0.974134097	0.98119932	AR
maple syrup urine disease type 1B	0.001373861	0.974952276	0.982183143	0.985422572	AR
Hb beta chain-related hemoglobinopathy	0.02332087	0.997286394	0.997286394	0.997286394	AR
USH2A-related disorders	0.013166316	0.990807489	0.991554557	0.992259857	AR
Pendred syndrome	0.008831553	0.995301778	0.99544166	0.99544166	AR
EVC-related Ellis-van Creveld syndrome	0.001493565	0.910665727	0.942743479	0.964976332	AR
hereditary fructose intolerance	0.008471328	0.991223706	0.99444873	0.996896982	AR
GRACILE syndrome	0.001382432	0.944586508	0.969598494	0.982703816	AR
AMT-related glycine encephalopathy	0.000965594	0.983451394	0.987672732	0.990823702	AR
aspartylglycosaminuria	0.000518162	0.941025641	0.963076923	0.967179487	AR
Cohen syndrome	0.003492704	0.957116622	0.966574575	0.971581577	AR
familial Mediterranean fever	0.010569842	0.998618614	0.998618614	0.998618614	AR
COL4A4-related Alport syndrome	0.001221021	0.970211282	0.975323899	0.979908312	AR
maple syrup urine disease type Ia	0.000890203	0.975383351	0.977764236	0.982653553	AR
tyrosinemia type I	0.002574575	0.987884739	0.988601921	0.990074707	AR
holocarboxylase synthetase deficiency	0.000645472	0.959215281	0.96210635	0.967888487	AR
Niemann-Pick disease, SMPD1-associated	0.002083224	0.985235217	0.988107863	0.990475994	AR
PPT1-related neuronal ceroid lipofuscinosis	0.002088087	0.950505472	0.960576102	0.964956535	AR
lipoamide dehydrogenase deficiency	0.000852999	0.873861436	0.962058459	0.985480339	AR
xeroderma pigmentosum group C	0.001013999	0.975202532	0.977962025	0.981772152	AR
ARSACS	0.002120428	0.981283801	0.983819355	0.986120407	AR
glycogen storage disease type Ia	0.002740341	0.990169224	0.990875023	0.992286622	AR
glycogen storage disease type Ib	0.001411544	0.990290701	0.990290701	0.991504363	AR
very long chain acyl-CoA dehydrogenase deficiency	0.004654822	0.989929472	0.993009193	0.994132224	AR
megalencephalic leukoencephalopathy with subcortical cysts	0.000643441	0.968033857	0.974944847	0.980550178	AR
hypophosphatasia, autosomal recessive	0.003055996	0.987793535	0.990339328	0.991995761	AR
alpha thalassemia	0.06490113	0.981831054	0.999274287	0.999274287	AR
Alstrom syndrome	0.003614301	0.978107489	0.980405785	0.982778761	AR
nephrotic syndrome, NPHS2-related	0.002589221	0.995304464	0.995743789	0.995743789	AR
Bardet-Biedl syndrome, BBS2-related	0.001328265	0.986983739	0.989410499	0.989410499	AR
COL4A3-related Alport syndrome	0.003268256	0.985026473	0.987197422	0.988957555	AR
CLN6-related neuronal ceroid lipofuscinosis	0.000262591	0.941585871	0.947741079	0.956310094	AR
Herlitz junctional epidermolysis bullosa, LAMA3-related	0.000521499	0.937738386	0.951156601	0.963422331	AR
Leigh syndrome, French-Canadian type	0.000708649	0.934263118	0.954213794	0.960183689	AR
11-beta-hydroxylase-deficient congenital adrenal hyperplasia	0.001404592	0.960159534	0.972803708	0.976425569	AR
D-bifunctional protein deficiency	0.001369273	0.930868096	0.957052004	0.983475399	AR
KCNJ11-related familial hyperinsulinism	0.0002344	0.941061428	0.95732954	0.965863632	AR
PCCB-related propionic acidemia	0.001718271	0.962991644	0.981640138	0.984111117	AR
Wilson disease	0.009594235	0.991442019	0.992182443	0.992773686	AR
Bardet-Biedl syndrome, BBS12-related	0.001243755	0.976869259	0.979831993	0.984008377	AR
PROP1-related combined pituitary hormone deficiency	0.001741994	0.994182463	0.994182463	0.99534597	AR
hexosaminidase A deficiency	0.003977713	0.9853024	0.991488981	0.992275714	AR
dysferlinopathy	0.003631159	0.97716252	0.979985776	0.982334897	AR
CLN3-related neuronal ceroid lipofuscinosis	0.002843615	0.988508327	0.993604994	0.995397115	AR
muscle-eye-brain disease	0.001467475	0.979022541	0.984958205	0.987648966	AR
EVC2-related Ellis-van Creveld syndrome	0.00157445	0.901250687	0.9330533	0.960825318	AR
xeroderma pigmentosum group A	0.000957864	0.956889437	0.977749387	0.985075808	AR
Fabry disease	0.000342619	0.918530093	0.918530093	0.918530093	X-linked
X-linked myotubular myopathy	4.77E-05	0.881091618	0.881091618	0.881091618	X-linked
Bardet-Biedl syndrome, BBS10-related	0.002790369	0.991311588	0.993196518	0.994861733	AR
congenital adrenal hyperplasia	0.019853702	0.978509933	0.978509933	0.978509933	AR
fragile X syndrome	0.005888129	0.985191948	0.98960861	0.992939624	X-linked
glutaric acidemia type 1	0.003406787	0.987834356	0.990976332	0.992433165	AR
Smith-Lemli-Opitz syndrome	0.016560818	0.994499551	0.996042297	0.997236806	AR
Andermann syndrome	0.000305782	0.905442275	0.925473866	0.951751867	AR
Usher syndrome type 3	0.000975111	0.987331506	0.990656783	0.990656783	AR
Fanconi anemia complementation group A	0.004784014	0.979097547	0.98244926	0.983863673	AR
6-pyruvoyl-tetrahydropterin synthase deficiency	0.000474506	0.960586429	0.96964613	0.96964613	AR
carbamoylphosphate synthetase I deficiency	0.000568463	0.967968966	0.975949016	0.982709892	AR
carnitine palmitoyltransferase IA deficiency	0.000478271	0.900631446	0.93379924	0.966967034	AR
tyrosinemia type II	0.000236836	0.507242058	0.838528938	0.892352626	AR
metachromatic leukodystrophy	0.004318609	0.985833271	0.990095613	0.991987571	AR
carnitine palmitoyltransferase II deficiency	0.005072644	0.985524876	0.991790265	0.995395611	AR
GJB2-related DFNB1 nonsyndromic hearing loss and deafness	0.024560353	0.998481345	0.998535003	0.998535003	AR
HADHA-related disorders	0.002644778	0.97644734	0.98562736	0.994936511	AR
familial dysautonomia	0.00173517	0.97636843	0.980336028	0.984129608	AR
X-linked Alport syndrome	0.000275681	0.976119403	0.976119403	0.976119403	X-linked
beta-sarcoglycanopathy	0.001080262	0.987286007	0.990299694	0.993972626	AR
polyglandular autoimmune syndrome type 1	0.003145693	0.991890433	0.992571503	0.993877817	AR
galactosemia	0.006830762	0.996569252	0.99717651	0.997674773	AR
X-linked congenital adrenal hypoplasia	4.61E-05	0.888667992	0.888667992	0.888667992	X-linked
spinal muscular atrophy	0.018850106	0.990237645	0.99940024	0.99940024	AR

Table S4: Clinical detection rate estimates when three reported cases are assumed to determine pathogenicity

mucolipidosis III gamma	0.000825854	0.918775848	0.940973005	0.976253854	AR
cystinosis	0.003213236	0.986112403	0.994610496	0.994610496	AR
USH1C-related disorders	0.001238128	0.984897778	0.987987526	0.988911562	AR
Bardet-Biedl syndrome, BBS1-related	0.005366863	0.994017167	0.996999491	0.997338942	AR
methylmalonic aciduria and homocystinuria, cblC type	0.005109334	0.9868441	0.99251869	0.994947353	AR
MYO7A-related disorders	0.004913812	0.99028436	0.991007328	0.991468491	AR
Sandhoff disease	0.003027014	0.891414169	0.941852266	0.967288283	AR
calpainopathy	0.005494707	0.991580744	0.992730011	0.993702971	AR
citrullinemia type 1	0.002025232	0.988918901	0.991187791	0.992869201	AR
Herlitz junctional epidermolysis bullosa, LAMC2-related	0.000774403	0.958513354	0.966516104	0.972751375	AR
GLDC-related glycine encephalopathy	0.001550012	0.983335183	0.986042383	0.988638633	AR
TGM1-related autosomal recessive congenital ichthyosis	0.003392383	0.978785056	0.985931367	0.9879366	AR
Pompe disease	0.011149681	0.991230702	0.992776217	0.993624492	AR
pycnodysostosis	0.000520649	0.954134315	0.983670856	0.987032738	AR
ATP7A-related disorders	4.19E-05	0.5	0.5	0.5	X-linked
MKS1-related disorders	0.001919276	0.990192331	0.992930947	0.994522768	AR
HMG-CoA lyase deficiency	0.000682821	0.877831282	0.877831282	0.877831282	AR
autosomal recessive osteopetrosis type 1	0.001821932	0.958835151	0.97716447	0.983274243	AR
dystrophinopathy (including Duchenne/Becker muscular dystrophy)	0.001121475	0.984745701	0.984745701	0.984745701	X-linked
ABCC8-related hyperinsulinism	0.002113378	0.986292993	0.987815994	0.987815994	AR
FKTN-related disorders	0.00120112	0.978155047	0.984095236	0.988432899	AR
X-linked juvenile retinoschisis	0.000121159	0.971084337	0.971084337	0.971084337	X-linked
biotinidase deficiency	0.004250571	0.994413788	0.994890176	0.995718183	AR
phenylalanine hydroxylase deficiency	0.019605948	0.996215078	0.996562259	0.996829472	AR
primary carnitine deficiency	0.004346014	0.989614109	0.992825986	0.993787085	AR
Northern epilepsy	0.000221691	0.953613281	0.953613281	0.97265625	AR
Canavan disease	0.001657308	0.974583656	0.986482128	0.992760734	AR
CLN5-related neuronal ceroid lipofuscinosis	0.000462236	0.979245841	0.981430489	0.98689211	AR
ataxia-telangiectasia	0.00317273	0.974115307	0.977170152	0.979878141	AR
isovaleric acidemia	0.001163564	0.914293563	0.950096145	0.966440802	AR
lipoid congenital adrenal hyperplasia	0.00077269	0.958863817	0.97819912	0.98546608	AR
Segawa syndrome	0.000669426	0.978944974	0.981430637	0.986889347	AR
methylmalonic acidemia, cblB type	0.00124321	0.981199272	0.990083132	0.992680407	AR
short chain acyl-CoA dehydrogenase deficiency	0.00547958	0.978050189	0.986691153	0.990683807	AR
PCCA-related propionic acidemia	0.001385767	0.965719195	0.973178368	0.97721772	AR
Krabbe disease	0.003769327	0.98693271	0.989920793	0.990964393	AR
congenital disorder of glycosylation type Ia	0.010637004	0.990220257	0.994084936	0.996720234	AR
congenital disorder of glycosylation type Ic	0.002506459	0.888725178	0.930678671	0.948962165	AR
congenital disorder of glycosylation type Ib	0.000705713	0.963343838	0.983745231	0.983745231	AR
pyruvate carboxylase deficiency	0.000208462	0.960703353	0.960703353	0.972663202	AR
methylmalonic acidemia, cblA type	0.00157035	0.755757392	0.844860784	0.931772694	AR
lysosomal acid lipase deficiency	0.002782208	0.975410295	0.988129554	0.993183649	AR
Nijmegen breakage syndrome	0.002101899	0.988480093	0.990261358	0.991857075	AR
ataxia with vitamin E deficiency	0.000935746	0.942241501	0.9654392	0.968315338	AR
PKHD1-related autosomal recessive polycystic kidney disease	0.00806609	0.981805607	0.985761433	0.987998707	AR
LAMA2-related muscular dystrophy	0.004340333	0.960097421	0.971511168	0.979602149	AR
primary hyperoxaluria type 3	0.005121511	0.985550698	0.98879981	0.991599857	AR
primary hyperoxaluria type 2	0.001398061	0.98863333	0.990079997	0.992559998	AR
primary hyperoxaluria type 1	0.003516707	0.992770136	0.994322113	0.995388502	AR
Gaucher disease	0.007484444	0.998170601	0.998475501	0.998475501	AR
Bloom syndrome	0.00276864	0.899458728	0.929639007	0.947343839	AR
Salla disease	0.00183288	0.961511904	0.979875708	0.991110475	AR
Joubert syndrome 2	0.000506866	0.986299904	0.989724928	0.989724928	AR
mucopolysaccharidosis type IIIB	0.00197876	0.963771558	0.979887646	0.986291498	AR
mucopolysaccharidosis type IIIC	0.001020071	0.948068772	0.965515299	0.973245843	AR
TPP1-related neuronal ceroid lipofuscinosis	0.002419113	0.99431916	0.99431916	0.99586848	AR
mucopolysaccharidosis type IIIA	0.003227229	0.992318061	0.993626657	0.995153351	AR
peroxisome biogenesis disorder type 3	0.001148436	0.988913833	0.990465896	0.992904853	AR
PCDH15-related disorders	0.001645422	0.983459533	0.984330084	0.984330084	AR
peroxisome biogenesis disorder type 6	0.000598462	0.965782743	0.973064676	0.977449496	AR
peroxisome biogenesis disorder type 4	0.000949901	0.982058736	0.982058736	0.985191338	AR
peroxisome biogenesis disorder type 5	0.000275476	0.984682888	0.984682888	0.988512166	AR
inclusion body myopathy 2	0.001667789	0.986853379	0.99020515	0.992103802	AR
cerebrotendinous xanthomatosis	0.002986308	0.90518436	0.939074175	0.962318121	AR
